# MEMS Ultrasound Transducers for Endoscopic Photoacoustic Imaging Applications

**DOI:** 10.3390/mi11100928

**Published:** 2020-10-12

**Authors:** Haoran Wang, Yifei Ma, Hao Yang, Huabei Jiang, Yingtao Ding, Huikai Xie

**Affiliations:** 1 Department of Electrical and Computer Engineering, University of Florida, Gainesville, FL 32611, USA; wanghaoran@ufl.edu; 2 School of Information and Electronics, Beijing Institute of Technology, Beijing 100081, China; 3120200697@bit.edu.cn (Y.M.); ytd@bit.edu.cn (Y.D.); 3 Department of Medical Engineering, University of South Florida, Tampa, FL 33620, USA; haoyang@usf.edu (H.Y.); hjiang1@usf.edu (H.J.)

**Keywords:** microelectromechanical systems, MEMS, ultrasound transducers, cMUT, pMUT, photoacoustic imaging, endoscopy, photoacoustic microscopy

## Abstract

Photoacoustic imaging (PAI) is drawing extensive attention and gaining rapid development as an emerging biomedical imaging technology because of its high spatial resolution, large imaging depth, and rich optical contrast. PAI has great potential applications in endoscopy, but the progress of endoscopic PAI was hindered by the challenges of manufacturing and assembling miniature imaging components. Over the last decade, microelectromechanical systems (MEMS) technology has greatly facilitated the development of photoacoustic endoscopes and extended the realm of applicability of the PAI. As the key component of photoacoustic endoscopes, micromachined ultrasound transducers (MUTs), including piezoelectric MUTs (pMUTs) and capacitive MUTs (cMUTs), have been developed and explored for endoscopic PAI applications. In this article, the recent progress of pMUTs (thickness extension mode and flexural vibration mode) and cMUTs are reviewed and discussed with their applications in endoscopic PAI. Current PAI endoscopes based on pMUTs and cMUTs are also introduced and compared. Finally, the remaining challenges and future directions of MEMS ultrasound transducers for endoscopic PAI applications are given.

## 1. Introduction

Photoacoustic imaging (PAI), also called optoacoustic imaging, is an emerging imaging technique that has been developed rapidly and has evolved from the laboratory to preclinical and clinical applications in the last decade [1,2,3]. Compared with traditional imaging techniques, such as X-ray computed tomography (CT), magnetic resonance imaging (MRI), and positron emission tomography (PET), PAI can achieve comparable resolution with smaller, faster, and less expensive systems [4,5]. Unlike pure ultrasound imaging that only reflects the mechanical properties of tissues with the image contrast provided by acoustic impedance variations, PAI possesses rich optical contrast and is capable of providing biofunctional or physiological parameters, such as oxygen saturation of hemoglobin, metabolic rate, and relative concentrations of water and lipid for noninvasive diagnosis of various diseases [6,7]. Compared to pure optical imaging techniques such as optical coherence tomography (OCT), confocal microscopy, and multiphoton microscopy [8,9], PAI can achieve a significantly larger imaging depth in most human tissues because the attenuation of the echo ultrasound signals in PAI is two to three orders of magnitude lower than that of the returning optical signals in those pure optical imaging modalities [9]. 

Thanks to the advantages described above, PAI has been extensively exploited in brain functional imaging [10,11], early cancer detection [12,13], vascular visualization [14,15,16], and diagnosis of arthritis [17,18]. Most of these efforts are based on benchtop PAI systems. Although PAI has a larger imaging depth compared to other optical “biopsy” techniques, e.g., OCT and multiphoton microscopy, the imaging depth of PAI is still limited to about 5 cm by the strong attenuation of the excitation laser in human tissues [19]. This means that PAI must be incorporated into endoscopic imaging systems so that it can be used to diagnose internal organs inside the human body. For example, PAI is an efficient technique for the early cancer detection of lung, colorectal, pancreatic, and prostate cancer [20,21,22]; however, for the in vivo diagnosis of internal organs, endoscopic PAI probes with small outer diameters (typically less than 5 mm) must be developed so that they can be delivered through esophageal or gastrointestinal tracts [23,24]. However, the development of endoscopic PAI is still at an early stage. One of the key challenges is miniaturizing the imaging components but still maintaining the imaging performance comparable to that of a benchtop PAI system.

**Figure 1:**
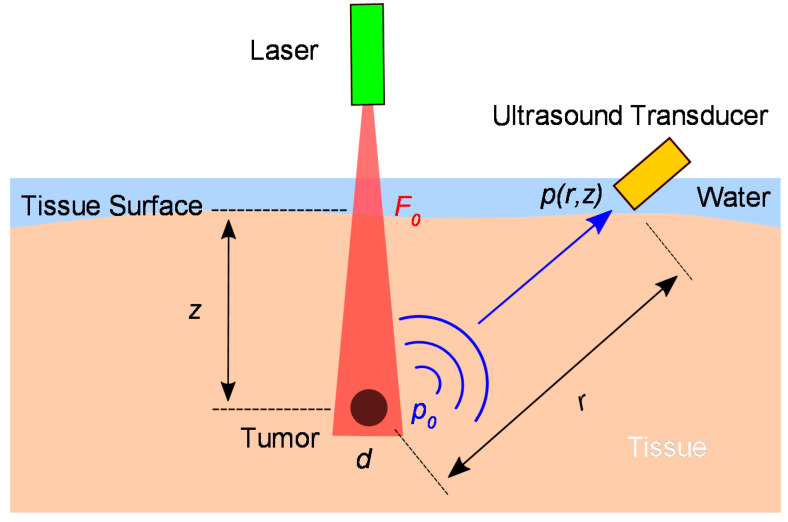
Diagram of laser illumination and photoacoustic wave detection in photoacoustic imaging (PAI).

The principle of PAI is illustrated in Figure 1, where a nanosecond pulsed laser is incident on a tissue sample with a fluence $F_{0}$ at the tissue surface. Assume there is a tumor located at a depth, z. Typically tumors exhibit significantly stronger optical absorption than normal tissue because angiogenesis in cancer development generates higher blood vessel densities in tumors [25]. Thus, the optical energy will be strongly absorbed by the tumor, leading to a higher temperature rise at the tumor site due to the photothermal effect. This temperature rise will cause thermal elastic expansion and generate a photoacoustic pulse with an initial pressure $p_{0}$, which will propagate in all directions and be detected by an ultrasound transducer placed at a distance of r from the tumor. An impedance-matching medium is typically employed to increase the acoustic coupling efficiency. For biological tissues, water is typically used since water has an acoustic impedance (~1.5 MRayl) similar to that of the various tissues (~1.6 MRayl). Assuming the tissue is deep (i.e., it can be considered semi-infinite to both the optical and acoustic waves), the peak pressure p(r,z) of the photoacoustic pulse reached to the ultrasound transducer can be expressed as [26]:

(1) $$p\left(r,z\right)=\frac{\Gamma }{4r}\mu_{a,tm}dF_{0}\exp\left(-\mu_{e,ts}z\right)\exp\left(-\mu_{ac,ts}r\right)$$

where Γ is the Gruneisen parameter representing the thermoacoustic efficiency, $\mu_{a,tm}$ is the optical absorption coefficient of the tumor, d is the effective diameter of the tumor, and $\mu_{ac,ts}$ is the coefficient of acoustic absorption of the tissue. $\mu_{e,ts}$ is the effective optical extinction coefficient of the tissue, which is defined as [3]:

(2) $$\mu_{e,ts}=\sqrt{3\mu_{a,ts}\left(\mu_{a,ts}+\mu_{s,\;ts}^{\prime }\right)}$$

where $\mu_{a,ts}$ and $\mu_{s,\;ts}^{\prime }$ are the optical absorption coefficient and effective scattering coefficient of the tissue, respectively.

By rewriting Equation (1), the maximum depth, $z_{max}$, that the tumor can be detected at is given by:

(3) $$z_{max}=-\frac{1}{\mu_{e,ts}}\ln\left[\frac{4p_{m\;}r}{\Gamma \mu_{a,tm}dF_{0}\exp\left(-\mu_{ac,ts}r\right)}\right]\;\;\;$$

where $p_{m}$ is the minimum detectable pressure by the ultrasound transducer, which is dependent on the sensitivity, bandwidth, and noise floor of the ultrasound transducer. As shown in Equation (3), the imaging depth is affected by multiple factors, including the incident laser pulse energy, the optical and acoustical properties of the tissue and tumor, and the performance of the ultrasound transducer. 

**Figure 2:**
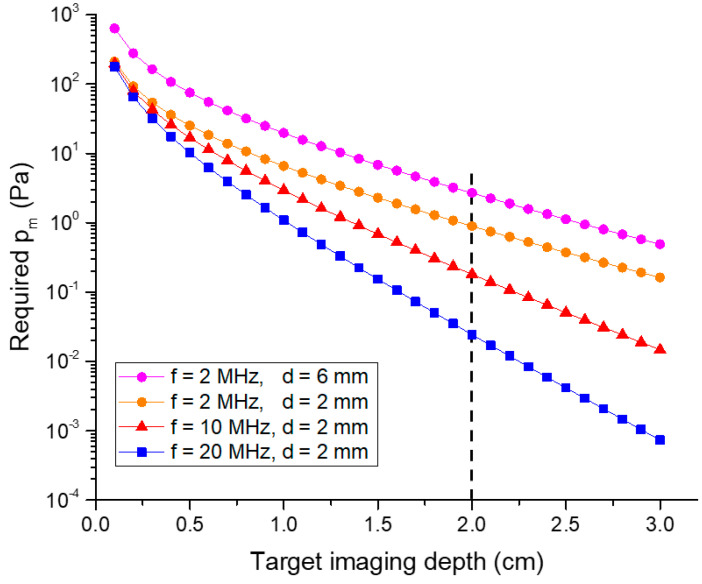
Required minimum detectable pressures of ultrasound transducers for different imaging depths for breast tissue with a 757 nm wavelength (note that this chart will vary with different tissues and/or different light wavelengths).

The required $p_{m}$ for a specific imaging depth of a given sample can be calculated using Equation (3) if a particular laser source is picked. Here, let us take the PAI of breast tumors as an example, which usually requires large imaging depth as most breast tumors are located 1 to 3 cm under the surface [27]. We assume $F_{0}=20$ mJ/cm^2^, which is within the safety limit given by the American National Standard Institute (e.g., 20 mJ/cm^2^ for a 532 nm wavelength and 100 mJ/cm^2^ for a 1064 nm wavelength [9]), and $\mu_{e,ts}\approx 1.1$ ${\mathrm{cm}}^{-1}$ for breast tissue with a 757 nm wavelength [12]. We can approximately estimate $\mu_{ac,ts}$ using $\mu_{ac,ts}=0.1f\;\left({\mathrm{cm}}^{-1}\right)$, where *f* is the bandwidth of the employed ultrasound transducer in MHz [26]. Both reported Γ and $\mu_{a,tm}$ have a wide range of values due to the heterogeneity of breast tissues and the availability of various laser wavelengths. The required $p_{m}$ versus imaging depth relation is plotted in Figure 2, assuming Γ=0.24 and $\mu_{a,tm}=0.1{\;\mathrm{cm}}^{-1}$ [12], which are the typical values for the laser wavelengths in the near-infrared (NIR) range. Tumor diameters of 2 mm and 6 mm are taken as an example to calculate the required $p_{m}$ for detecting small breast tumors and show the effect of the tumor size according to [28]. As shown in Figure 2, to image 2 cm deep, 2 mm diameter tumors, the required $p_{m}$ levels are about 0.89 Pa, 0.18 Pa, and 0.02 Pa for ultrasound transducers with a center frequency of 2 MHz, 10 MHz and 20 MHz, respectively.

Since $p_{m}$ is related to the noise spectrum in the bandwidth of interest, the noise equivalent pressure (NEP) is also used to quantify the minimum detectable pressure of an ultrasound transducer, which can be expressed as a spectral density with units of $\mathrm{Pa}/\sqrt{\mathrm{Hz}}$. NEP varied over a wide range, depending on the transducer material, active detection area *A*, center frequency f, and preamplifier design. Typical NEP values of commercially available piezoelectric ultrasound transducers are reported in [29], e.g., 4.6 $\mu \mathrm{Pa}/\sqrt{\mathrm{Hz}}$ (A=1439 mm^2^, f=2.45 MHz, FerroPerm Piezoceramics) and 200 $\mu \mathrm{Pa}/\sqrt{\mathrm{Hz}}$ (A=30 mm^2^, f=50 MHz, Olympus NDT). 

The spatial resolution of the PAI can be either acoustically determined or optically determined, depending on whether an optical or acoustic focusing technique is used [2]. For the acoustically determined resolution, the axial imaging resolution $R_{A}$ and lateral imaging resolution $R_{L}$ can be expressed as [30]:

(4) $$R_{A}=\frac{0.88\;c}{\Delta f}\;$$

(5) $$R_{L}=\frac{0.71\;c}{f\;NA}\;$$

where c is the acoustic speed in the tissue and f, Δf, and NA are the acoustic center frequency, bandwidth, and numerical aperture of the ultrasound transducer, respectively.

Based on Equations (3)–(5), it can be seen that ultrasound transducers are the most critical components in PAI systems, as their NEP, bandwidth, and center frequency will directly determine the signal-to-noise ratio (SNR), spatial resolution, and imaging depth of a PAI system. Current commercial ultrasound transducers that have been widely used in benchtop PAI systems are dominated by bulk piezoelectric transducers [27,31,32]. These bulk ultrasound transducers, referred to as conventional ultrasound transducers in this article, consist of piezoelectric plates or disks that are poled along the thickness direction and work on the thickness extension mode. In this mode of operation, the center frequency of an ultrasound transducer is directly determined by the thickness of the piezoelectric element, with the fundamental resonance frequency occurring when the thickness is equal to half the wavelength of the acoustic wave in the piezoelectric material [33]. However, conventional ultrasound transducers are difficult and expensive to be fabricated into arrays for high-frequency and 3D volumetric imaging applications, which require very small kerfs (the separation trench between two adjacent elements) to reduce crosstalks and avoid grating lobes as high-frequency arrays have small pitches [34,35]. Very thin ceramic sheets are also required to achieve high frequencies. For example, the required resonance frequency of ultrasound transducers for high-resolution PAI applications may be as high as over 50 MHz [36,37], which means the thickness of the piezoelectric element must be less than 45 μm if PZT-5H is used.

Fortunately, microelectromechanical systems (MEMS) technology can provide a solution to many of the challenges facing endoscopic PAI [38,39]. With advantages of small size, low cost with batch fabrication, high performance, and easy integration with electronics, MEMS, or micromachined ultrasound transducers (MUTs) have drawn more and more interest for endoscopic PAI applications. There are mainly two types of MUTs: piezoelectric MUTs (pMUTs) and capacitive MUTs (cMUTs). Several types of MUTs based PAI endoscopes have been developed for various applications such as human gastrointestinal tract imaging [40], intravascular spectroscopic PAI [41], visceral pathological changes screening [42], and breast tumor margins evaluation [43]. Dual-modality endoscopes with integrated ultrasound and photoacoustic imaging abilities were also developed for intravascular imaging and atherosclerosis diagnosis [44,45]. Probe diameter and imaging resolution, depth, and speed are the key parameters of an endoscopic PAI system, which are largely dependent on the form factor and characteristics of the employed MUT. There are all kinds of tradeoffs among those parameters. Generally, MUTs with a large sensing area will help increase the SNR and thus the imaging depth as well, but will make the probe diameter larger. High-frequency MUTs will increase the imaging resolution, but the cost is the reduced imaging depth due to the higher acoustic attenuation. For instance, some PAI endoscopes may have achieved good spatial resolution with high-frequency MUTs, but they may suffer from either insufficient imaging depth or slow imaging speed [46,47]. Developing MUT arrays with high element density will increase the imaging speed, but will face the challenges of large probe size and increased complexity of the interface electronics. Vast research efforts have been invested in developing better MUTs and thus better endoscopic PAI systems and enormous progress has been made, which is evidenced by a large amount of literature produced [48,49,50]. In order to better leverage the existing achievements, it is necessary to review and organize the MUTs developed up to date and their applications in endoscopic PAI systems, compare their advantages and limitations, and point out future directions with respect to these challenges. 

There are several reviews on various aspects of PAI- and MEMS-based endoscopic imaging technologies. Manwar et al. presented an overview of ultrasound detection technologies for PAI [51]. Lee et al. reviewed a number of MEMS technologies for PAI, including MEMS scanning mirrors, several types of MEMS-based detectors including MUTs, microring resonators, and micromachined silicon acoustic delay lines and multiplexer [38]. Chan et al. presented a review of PAI based on cMUTs [52]. Qiu et al. reviewed MEMS sensors and actuators for various fiber-optic endoscopic imaging modalities such as fluorescence imaging, OCT, confocal imaging, PAI, and two-photon imaging [53]. Both MUTs and endoscopic PAI were discussed in these articles, but MUTs-based endoscopic PAI were not reviewed thoroughly. 

In this review, we look into all of the representative MUT designs specifically for endoscopic PAI systems, summarize the recent progress of MUTs and MUTs-based endoscopic PAI, and analyze their limitations and potential solutions for high-resolution, high-speed endoscopic PAI at large penetration depths. Over 150 articles about MUTs design, fabrication, and application in endoscopic PAI have been reviewed. Both pMUTs and cMUTs are discussed. Since pMUTs are more extensively used than cMUTs in endoscopic PAI systems, we focus attention on pMUTs in this article. This review is organized as follows. In Section 2, we review pMUTs, including both thickness extension mode pMUTs and flexural vibration mode pMUTs. We will focus more on flexural vibration mode pMUTs, and review their materials, fabrication, modeling, sensitivity and bandwidth enhancement, and endoscopic PAI applications. In Section 3, we introduce the cMUT operation principle, the state of the art of cMUTs, and their applications in endoscopic PAI. In Section 4, we compare endoscopic PAI systems based on pMUTs and cMUTs. In Section 5, we summarize the review and discuss future directions of MUTs for endoscopic PAI applications. 

## 2. pMUTs and Their Endoscopic PAI Applications

In principle, pMUTs can work as ultrasound receivers or transmitters, operating on the direct and converse piezoelectric effects, which are governed by the piezoelectric constitutive equations, i.e.,

(6) $$D=d\;T+\varepsilon^{T}E$$

(7) $$S=s^{E}T+d^{t}E$$

where *D* is the electrical displacement, *T* is the mechanical stress, *E* is the electrical field, *S* is the strain, $\varepsilon^{T}$ is the dielectric constant measured at a constant stress, $s^{E}$ is the elastic compliance measured at a constant electrical field, and *d* and $d^{t}$ are the piezoelectric coefficients for the direct and converse piezoelectric effects, respectively.

The piezoelectric coefficients are third-rank tensors with respect to the crystal orientation and the poling direction. In analytical approaches, the matrix equations can be simplified and only the piezoelectric constants $d_{31}$ and $d_{33}$ are widely used to evaluate piezoelectric materials’ properties and calculate the responses of pMUTs.

pMUTs can be classified into two types: thickness extension mode pMUTs and flexural vibration mode pMUTs, corresponding to the piezoelectric modes represented by $d_{33}$ and $d_{31}$, respectively. These two types of pMUTs and their applications in endoscopic PAI will be introduced in the following sections. 

### 2.1. Thickness Extension Mode pMUTs

#### 2.1.1. Working Principle

**Figure 3:**
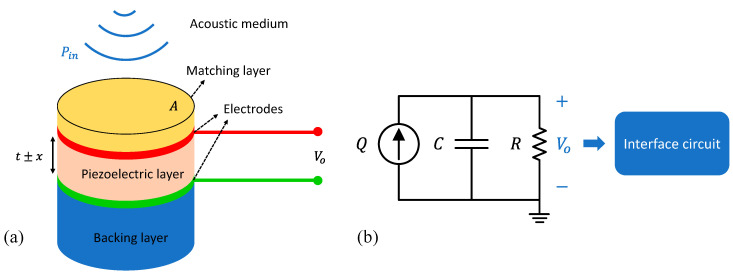
(**a**) Three-dimensional (3D) schematic of the typical structure of a thickness extension mode pMUT receiver. (**b**) Electrical model of the pMUT receiver.

The typical structure of a thickness extension mode pMUT receiver is shown in Figure 3a, which consists of a piezoelectric layer sandwiched between a top and bottom electrode, a backing layer, and a front acoustic matching layer. The piezoelectric layer is poled along its thickness direction and its thickness, *t,* determines the resonance frequency, *f,* of the pMUT as [54]:

(8) $$f=n\frac{c_{p}}{2t}$$

where *n* is an odd integer, $c_{p}$ is the sound speed in the piezoelectric layer. The fundamental resonance frequency occurs when n=1. For a typical 5 MHz fundamental frequency for PAI applications, the thickness of the piezoelectric layer is in the order of 200–700 μm depending on the actual piezoelectric materials employed. Thus, bulk piezoelectric materials are normally needed to manufacture thickness extension mode pMUTs. Table 1 lists the properties of commonly used bulk piezoelectric materials, including polyvinylidene fluoride (PVDF), single-crystal lead magnesium niobite-lead titanate (PMN-PT), single-crystal lithium niobite (LiNbO_3_), and ceramic lead zirconate titanate (PZT).

Due to the large acoustic impedance mismatch between the piezoelectric element (~34 MRayl for PZT) and the load medium (~400 Rayl for air, ~1.5 MRayl for water), a front acoustic matching layer is usually employed for improving the energy transmission between the load medium and the active layer. In theory, the maximum transmission is achieved when the front acoustic matching layer is designed as [54]:

(9) $$t_{m}=\frac{\lambda_{m}}{4}$$

(10) $$Z_{m}=\sqrt{Z_{p}Z_{l}}$$

where $t_{m}$ is the thickness of the matching layer, $\lambda_{m}$ is the acoustic wavelength in the matching layer, and $Z_{m}$, $Z_{p}$, and $Z_{l}$ are the acoustic impedances of the matching layer, piezoelectric layer, and loading medium, respectively. The backing layer works not only as a mechanical support to the piezoelectric layer, but also helps to absorb the acoustic energy and damp out the ring-down vibrations.

As shown in Figure 3a, when an incoming ultrasound wave $P_{in}$ strikes the pMUT with a sensing area of *A*, the piezoelectric layer will be deformed with a displacement *x*, thus generating charges due to the $d_{33}$ piezoelectric effect and inducing a voltage. The electrical model of the pMUT receiver is shown in Figure 3b, where the pMUT is modeled as a charge source Q, with a capacitor *C* and a dielectric loss *R* connected in parallel. The voltage output $V_{o}$ is then picked up by an interface circuit for signal processing. The noises in a pMUT receiver may originate from the acoustic medium, the piezoelectric element, and the interface circuit. The thermal noise spectral density (in $V^{2}/\mathrm{Hz}$) from the acoustic medium is given by [58]:

(11) $$v_{am}^{2}=4k_{B}TAZ_{a}{\left|\frac{G}{2A}\right|}^{2}=G^{2}\frac{k_{B}TZ_{a}}{A}$$

where $Z_{a}$ is the specific acoustic impedance of the medium (in Rayl), $k_{B}$ is the Boltzmann constant, and *T* is the absolute temperature of the medium. *G* represents the ratio between the pMUT output voltage and the incident pressure (in V/Pa), which is related to the piezoelectric constant $d_{33}$, thickness t, and clamped relative permittivity $\varepsilon_{33,r}^{S}$ of the piezoelectric material in the pMUT, i.e., [59]

(12) $$G=\frac{V_{o}}{P_{in}}=-\frac{d_{33}t}{\varepsilon_{0}\varepsilon_{33,r}^{S}}$$

where $\varepsilon_{0}=8.85\times 10^{-12}\;F/m$ is the vacuum permittivity. The noise of the piezoelectric element can be modeled as the thermal noise of a resistor *R* in parallel with a capacitor *C*. The noise spectral density over the bandwidth Δf is given by:

(13) $$v_{pe}^{2}=4k_{B}TR\Delta f=4k_{B}TR\frac{1}{4RC}=\frac{k_{B}T}{C}$$

With a matched impedance, the additional noise from the interface circuit can be described by its noise factor $F_{n}$, which is the ratio of the circuit noise referred to the input of the interface circuit to the noise of the piezoelectric element [29], i.e.,

(14) $$v_{ic}^{2}=v_{pe}^{2}\left(F_{n}-1\right)$$

Thus, $p_{m}$ and *NEP* can be expressed as:

(15) $$p_{m}=\frac{\sqrt{v_{am}^{2}\left(f\right)\Delta f+v_{pe}^{2}+v_{ic}^{2}}}{G}$$

(16) $$NEP\left(f\right)=p_{m}/\sqrt{\Delta f}$$

Therefore, we can calculate the $p_{m}$ and NEP values of thickness extension mode pMUTs based on different piezoelectric materials. The results are listed in Table 2, where we still consider a 5 MHz center frequency and a 2 MHz bandwidth. We also assume the pMUT area *A* is 30 mm^2^, $Z_{a}=1.5\times 10^{6}$ Rayl for water, and $F_{n}=2$, which is reasonable for most low-noise interface circuits [29]. Table 2 gives a general comparison of the NEP and *p_m_* values among thickness extension mode pMUTs based on different materials but keeping the frequency, bandwidth, and sensing area the same. In the following, we will review the thickness extension mode pMUTs in terms of NEP from high to low. 

#### 2.1.2. Thickness Extension Mode pMUTs for Endoscopic PAI Applications

Various thickness extension mode (TEM) pMUTs have been developed and applied in endoscopic PAI based on different piezoelectric materials, including PVDF [60,61,62], LiNbO_3_ [40,63,64,65,66], PZT [24,41,43,46,67,68,69], and PMN-PT [70,71,72]. Progress has been made in reducing pMUT size, improving pMUT sensitivity, and achieving high-resolution PAI by using different piezoelectric materials and fabricating high-frequency pMUTs and focused pMUTs. In this section, we will review these TEM pMUTs based on the types of piezoelectric materials employed and their applications in endoscopic PAI applications. The focused TEM pMUTs in endoscopic PAI are reviewed at last.

##### PVDF-Based TEM pMUTs

PVDF is a piezoelectric polymer that has been explored as an ultrasound transducer material for over 40 years. PVDF has a low acoustic impedance (2.7–4 MRayl), which facilitates impedance-matching to human tissue or commonly used ultrasound gels, and exhibits a broad bandwidth. Kim et al. presented a 25 MHz PVDF ultrasonic transducer array with a measured −6 dB bandwidth of 100% [73]. Moreover, PVDF possesses a high degree of flexibility, which can be easily fabricated into convex shapes as focused ultrasound transducers without the need of acoustic lenses. Since, in 1990, Mo et al. reported the first micromachined PVDF ultrasonic transducer [74], several PVDF pMUTs have been developed with applications in pulse-echo ultrasound [75], minimally invasive imaging [76], and mechanical properties’ measurement [77]. However, the low dielectric constant of PVDF makes it susceptible to parasitic capacitances and the miniaturization usually causes an electrical impedance mismatch to the typical cables, which require high-input impedance amplifiers in close proximity to transducers or integration with electronics. In 2010, Chandrana et al. designed PVDF pMUTs and analyzed their parasitic capacitances, showing that on-chip parasitic capacitances degraded the performances of transducers and can be reduced by using polycarbonate instead of silicon substrates [78]. Furthermore, PVDF’s small piezoelectric constant and low electromechanical coupling coefficient are limitations of PVDF pMUTs, leading to low sensitivity. 

Thickness extension mode PVDF pMUTs have been reported for PAI applications [79,80,81,82]. Zangabad et al. reported a kerfless PVDF transducer array of 10 elements with dimensions of 1 mm × 1.5 mm × 28 μm for PAI, showing a $p_{m}$ of 170 Pa over the entire bandwidth (10 Hz–10 MHz) [80]. Daeichin et al. present a PVDF ultrasound transducer with dimensions of 0.6 mm (w) × 0.6 mm (h) × 52 μm (t) with an integrated readout circuit for intravascular PAI, resulting in a $p_{m}$ of 30 Pa over the entire bandwidth (1–20 MHz) [81]. As shown in Figure 2, this level of sensitivity can only image a depth of less than 0.5 cm. Although a variable thickness multilayered PVDF transducer is proposed to improve the sensitivity and bandwidth for PAI applications, the achieved sensitivity is still not comparable with those of PZT-based ultrasound transducers [82]. 

**Figure 4:**
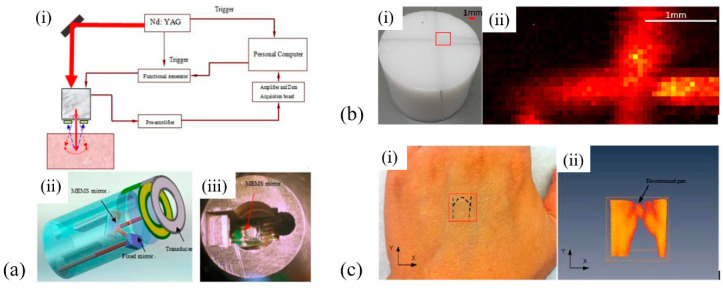
(**a**) Microelectromechanical systems (MEMS)-based photoacoustic probe: schematic of the system (a-i), 3D rendering (a-ii), and photograph (a-iii) of the scanning probe. (**b**) Photograph (b-i) and recovered photoacoustic image (b-ii) of the phantom. (**c**) Photograph of the hand (c-i) with a recovered 3D photoacoustic image of blood vessels (c-ii) (Reproduced with permission from OSA [60]).

In 2010, Xi et al. developed a MEMS-based photoacoustic imaging system with a MEMS scanning mirror and a ring-shaped PVDF transducer integrated into a miniature probe with an outer diameter of 11.5 mm [60] (Figure 4a). The ring-shaped, single-element PVDF ultrasound transducer with a center frequency of 2.5 MHz is fabricated using a 110 μm thick, Ag ink-printed PVDF film. Photoacoustic images of pencil lead embedded in an agar phantom (Figure 4b) and blood vessels under the skin of a human hand (Figure 4c) have been successfully demonstrated using this imaging system, showing an imaging depth of up to 2.5 mm and a lateral resolution of about 0.7 mm. 

##### LiNbO_3_-Based TEM pMUTs

**Figure 5:**
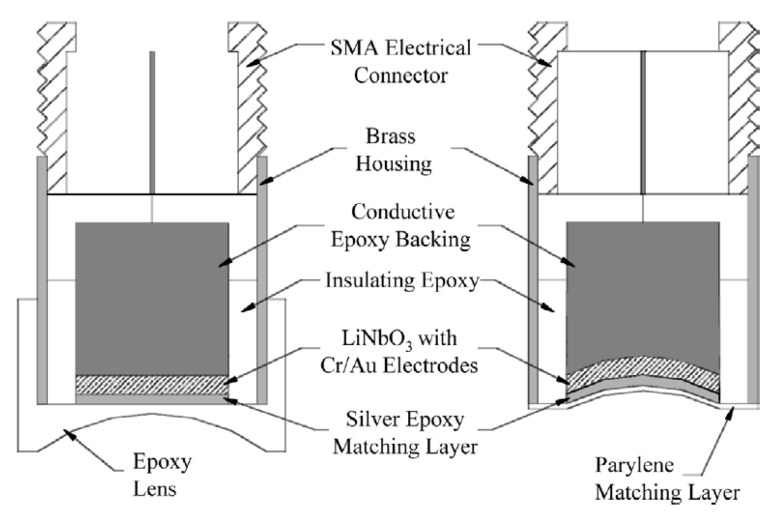
Design cross-sections of lens-focused (left) and press-focused (right) LiNbO_3_-based pMUTs (Reproduced with permission from IEEE [55]).

As shown in Table 1, LiNbO_3_ displays electromechanical coupling that was three times greater than PVDF. In 2002, Snook et al. fabricated high-frequency, single-element transducers based on different materials with the same 3 mm aperture size and near 50 MHz center frequency, showing that LiNbO_3_ pMUTs had a significantly improved sensitivity than PVDF pMUTs [83]. As a lead-free single crystal with a relatively high $k_{t}$ and a small dielectric constant, LiNbO_3_ is a good candidate for fabricating lead-free ultrasound transducers. Its high acoustic wave velocity also makes it a good choice for high-frequency thickness extension mode pMUTs. For example, in 2003, Cannata et al. presented the design, fabrication, and testing of single-element LiNbO_3_ pMUTs for medical imaging applications [55]. They use 36° Y-cut LiNbO_3_ wafers with thicknesses of 90 μm, 60 μm, and 30 μm to fabricate pMUTs operating at 25 MHz, 50 MHz, and 80 MHz, respectively. Acoustic focusing is also implemented using an acoustic lens made of epoxy and a curved LiNbO_3_ layer formed by high pressure, respectively, both of which are shown in Figure 5. The measurements of the fabricated transducers show the center frequencies in the 20–80 MHz range with −6 dB fractional bandwidths ranging from 57% to 74%. Although LiNbO_3_ has a good electromechanical coupling, its high acoustic impedance and very high electrical and mechanical quality factor make it difficult to design pMUTs with broad bandwidths [55]. By producing an inversion layer without changing the overall thickness of the piezoelectric layer, even-order higher frequency can be generated and broad bandwidth can be achieved. In 2005, Zhou et al. reported a 60 MHz LiNbO_3_ pMUT with a −6 dB bandwidth of 80% using a half-thickness inversion layer and two matching layers [84]. In addition to the planar inversion layer, in 2012, Chen et al. presented a half-concaved 60 MHz LiNbO_3_ pMUT with a curved inversion layer [85]. By using the mechanic dimpling technique to achieve a half-concaved structure with the continuous change of thickness, a broad −6 dB bandwidth of 123% was achieved without a matching layer. 

**Figure 6:**
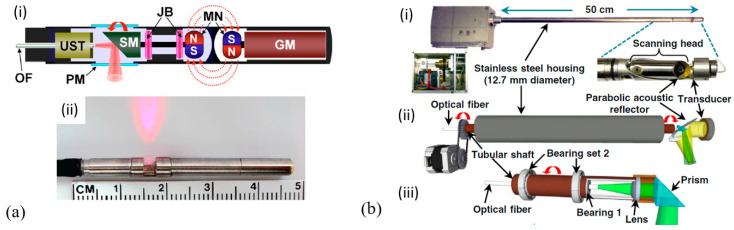
(**a**) Schematic (a-i) and photograph (a-ii) of the developed photoacoustic endoscopic probe. GM: geared micromotor, JB: jewel bearings, MN: magnets, OF: optical fiber, PM: plastic membrane, SM: scanning mirror, UST: ultrasonic transducer (Reproduced with permission from OSA [63]). (**b**) Photograph (b-i) and schematic (b-ii) of the developed photoacoustic probe, and schematic of the rotary junction and light guiding optics (b-iii) (Reproduced with permission from OSA [65]).

LiNbO_3_ pMUTs for endoscopic PAI applications have been demonstrated [63,65,86]. In 2009, Yang et al. developed a miniaturized imaging probe with a diameter of 4.2 mm based on a single-element 43 MHz LiNbO_3_ pMUT for photoacoustic endoscopy [63]. Figure 6a shows a schematic of the proposed endoscopic probe, where a scanning mirror is controlled by a mechanical micromotor at a rotational speed of 2.6 Hz to achieve a B-scan. The performance of the system was determined by imaging a carbon fiber in clear and turbid media, showing that the transverse resolution degraded from 230 μm to 450 μm and 177 μm to 520 μm with the target placed from the probe surface to 2.9 mm and 1.9 mm depths in the clear medium and the turbid medium, respectively. B-scan imaging of the biological tissues of a rat was also demonstrated. For clinical applications, a higher scanning speed is required. By switching the locations of the pMUT and mechanical actuation source, the space restriction for the motor was eliminated at the distal end thereby enabling the use of more powerful motors for faster scanning. Based on this configuration, in 2014, Li et al. developed a 12.7 mm diameter dual-mode photoacoustic and ultrasound rigid probe with a scanning speed exceeding 20 Hz for human urogenital imaging applications [65] (Figure 6b). With a single-element 40 MHz LiNbO_3_ pMUT used, deep-tissue imaging was demonstrated by imaging a black needle inserted into the leg of a rat to a depth of more than 5 mm. 

##### PZT-Based TEM pMUTs

**Figure 7:**
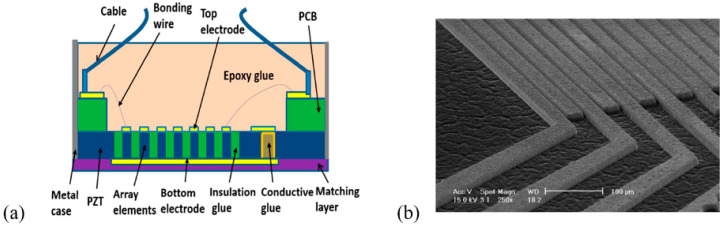
(**a**) Schematic of a PZT-based linear pMUT array (Reproduced with permission from MDPI [94]). (**b**) SEM image of a linear array dry-etched from a PZT composite film (Reproduced with permission from IEEE [93]).

PZT ceramics or composites have much higher $d_{33}$ than LiNbO_3_, and thus have been the dominant piezoelectric materials for fabricating ultrasound transducers and transducer arrays for several decades [87,88]. The relatively large dielectric constant of PZT ceramics also makes it easier to match the electrical impedance of conventional electronics, especially for pMUTs with the small element size. Single-element pMUTs and linear pMUT arrays based on PZT have been developed [89,90,91,92,93,94]. For example, in 2005, Vos et al. reported a single-element pMUT based on PZT ceramic for harmonic intravascular ultrasound imaging [90]. The PZT disk was thinned down to a thickness of about 70 μm for a resonance frequency of 20 MHz. The measurement of the transmission transfer function showed peaks at 22 MHz and 40 MHz, with a −6 dB bandwidth of 30% and 25%, respectively. Lukacs et al. developed a 30 MHz, 64-element linear array based on laser micromachining, which offers advantages of smaller kerfs and more intricate kerf designs than conventional “dice-and fill” methods [91]. However, PZT ceramics have large grain size in the order of 5–10 μm, which are not suitable for high-frequency (>50 MHz) pMUTs as the required thickness of the PZT can be on the order of tens of micrometers [54]. Moreover, it is very difficult and time-consuming to thin down PZT ceramics to such small thicknesses. PZT thick-film technology provides a solution to this problem. By using a modified sol–gel method with PZT ceramic powders mixed into the sol–gel precursor solution, PZT composite thick films (>10 μm) can be produced for fabricating high-frequency pMUTs [54]. In 2006, Zhang et al. reported a 103 MHz pMUT with a −6 dB bandwidth of 70% based on a 13 μm thick PZT composite film deposited by a modified sol–gel method, which is suitable for high-frequency imaging applications at the cellular level [92]. In 2010, Zhou et al. presented an 80 MHz linear pMUT array with 32 elements based on a 20 μm thick, spin-coated PZT composite film [93]. Small kerfs (12 μm) in the array were formed by reactive ion etching (RIE) and filling with epoxy, which has advantages over mechanical dicing and laser micromachining techniques in the bulk production of miniature devices at one time. Figure 7 shows the schematic of a PZT-based linear pMUT array and an SEM image of a linear array dry-etched from a PZT composite film. 

One of the applications of PZT pMUTs is PAI, especially in endoscopic settings. Dangi et al. developed a thickness mode pMUT based on a 150 μm thick PZT ceramic for PAI applications [95]. The pMUT has an outer diameter of 2.5 mm with a central hole where a 0.4 mm diameter multimode optical fiber is integrated for light delivery. The measured center frequency and −6 dB fractional bandwidth are 13.1 MHz and 30%, respectively. Yang et al. developed a 20 MHz pMUT (1.5 mm × 3 mm) based on PZT ceramic for PAI, showing a −6 dB bandwidth of 63% and a NEP of 0.24 $\mathrm{mPa}/\sqrt{\mathrm{Hz}}$ at 20 MHz [96]. Both of them can be potentially applied in photoacoustic endoscopes.

In 2012, Xi et al. developed an 11.5 mm diameter intraoperative photoacoustic imaging probe based on a 5.5 MHz, ring-shaped PZT ceramic-based pMUT and a MEMS scanning mirror for the evaluation of breast tumor margins [43]. The imaging ability was demonstrated by accurately and three-dimensionally mapping tumors then inspecting the completeness of tumor resection during surgery in a tumor-bearing mouse model, in which a penetration depth of 2.3 mm was achieved. 

**Figure 8:**
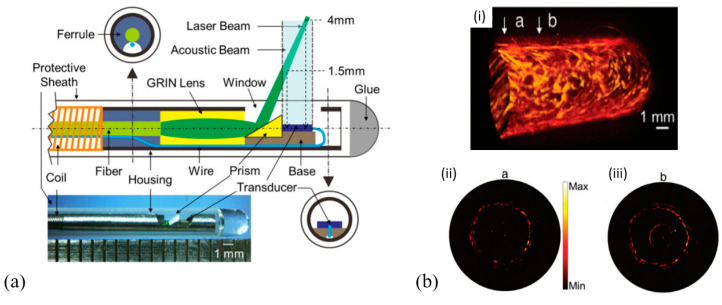
(**a**) Schematic (top) and photograph (bottom) of the photoacoustic/ultrasonic dual-modality endoscope. (**b**) Three-dimensional (3D) photoacoustic image of the rat’s rectum (b-i), and B-scan photoacoustic images of the “a” (b-ii) and “b” (b-iii) cross-sections (Reproduced with permission from John Wiley and Sons [24]).

In 2018, Li et al. presented a photoacoustic/ultrasonic dual-modality endoscope based on a rotary joint controlled torque coil scanning and a single-element PZT composite-based pMUT (0.6 × 0.5 × 0.2 mm^3^, 40 MHz, 60% fractional bandwidth, Blatek, USA), which can provide a full 360° field of view for gastrointestinal tract imaging applications [24]. The endoscope has a diameter of 2.5 mm. The schematic and a photo of the designed endoscope are shown in Figure 8a. The photoacoustic imaging performance of this endoscope is evaluated with phantoms and in vivo animal studies. A carbon fiber (~10 µm diameter) is imaged with a distance of 1.5 mm to 4.5 mm from the center of the endoscope, showing an SNR of higher than 20 dB in the imaging depth range of 4 mm. The transverse and axial resolutions at 2.7 mm are 254 μm and 49 μm, respectively. Using this system, 3D endoscopic PAI of a rat’s colorectum has been demonstrated (Figure 8b).

By using the same PZT composite-based pMUT, photoacoustic endoscopes with smaller sizes were demonstrated. The same group developed catheters for intravascular photoacoustic imaging with diameters of 1.1 mm and 0.9 mm [41,42]. Moreover, Li et al. integrated photoacoustic microscopy and white-light microscopy into a 1.7 mm diameter miniature probe for image guidance, where the same 40 MHz pMUT from Blatek was employed [67]. In this work, by coupling two lasers (wavelength of 532 nm and 680 nm) into one single core of the imaging bundle, scalable lateral resolution and imaging depth were also achieved. The best resolutions were determined to be 7.2 μm (532 nm) and 12.2 μm (680 nm), while the maximum imaging depths were 0.61 mm (532 nm) and 7.2 mm (680 nm) determined by visualizing a needle inserted into the chicken breast with SNRs > 20 dB. 

##### PMN-PT-Based TEM pMUTs

**Figure 9:**
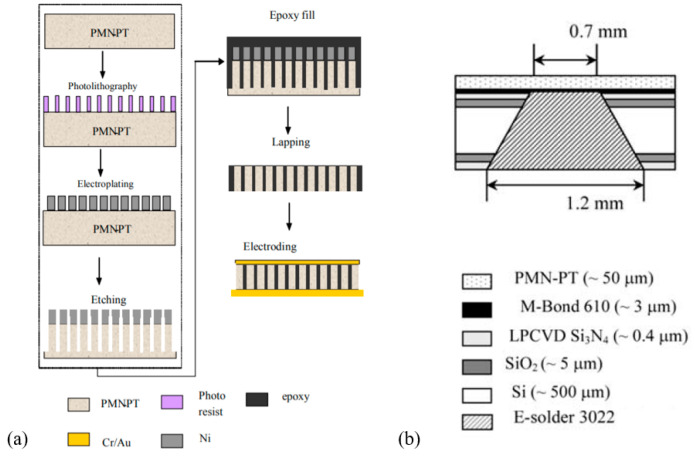
(**a**) Process flow for fabricating PMN-PT/epoxy 1-3 composite-based pMUT (Reproduced with permission from IEEE [98]). (**b**) Cross-view schematic of a PMN-PT single-crystal-based pMUT (Reproduced with permission from Springer Nature [100]).

PMN-PT has an even higher $d_{33}$ and electromechanical coupling. With the breakthrough of discovering ultrahigh coupling and piezoelectric properties in single-crystal PMN-PT in 1989, efforts have been made to develop high-performance transducers using PMN-PT [97]. In 2006, Jiang et al. fabricated high-frequency (20–50 MHz) pMUTs based on a PMN-PT single-crystal/epoxy 1-3 composite, showing a high electromechanical coupling coefficient of 0.72 [98]. The PMN-PT composite was obtained by using deep dry etching of a PMN-PT single-crystal wafer to form deep trenches, filling the trenches with epoxy, and then being lapped down to the desired thickness, as shown in Figure 9a. In 2007, Zhou et al. developed a high-frequency ultrasonic needle transducer based on single-crystal PMN-PT with an aperture size of only 0.4 mm; the measured center frequency and −6 dB fractional bandwidth were 44 MHz and 45%, respectively [99]. In 2010, by using a hybrid method involving mechanical grinding and wet etching, Peng et al. successfully thinned down a PMN-PT single-crystal wafer bonded on a silicon wafer to around 50 μm and demonstrated a pMUT with a center frequency of 35 MHz and a −6 dB fractional bandwidth of 34% [100]. The fabricated PMN-PT pMUT had a chip size of 1.5 mm × 1.5 mm; its cross-sectional view is shown in Figure 9b. In 2017, by lapping and mechanically dimpling single-crystal PMN-PT, Fei et al. fabricated a 35 MHz half-concave-shaped focused pMUT for intravascular ultrasound imaging applications [101]. Owing to the dimpling process, the thickness changes continuously along the curved surface, leading to multiple resonances around the center frequency, thus resulting in an improved −6 dB bandwidth of 54% compared to 28% for a flat PMN-PT pMUT fabricated using the same process. The spatial resolution of the pMUT was also improved. 

**Figure 10:**
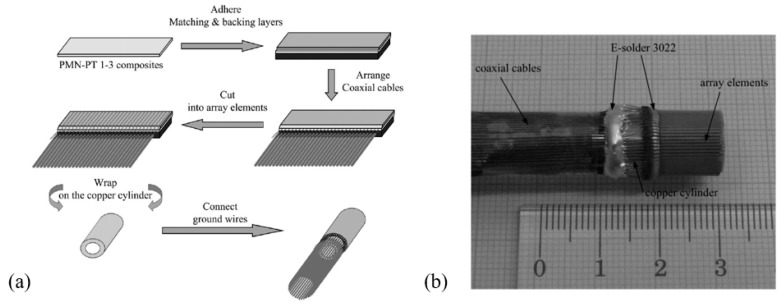
Fabrication process (**a**) and photograph (**b**) of a PMN-PT/epoxy 1-3 composite-based radial pMUT array (Reproduced with permission from IEEE [102]).

Compared with single-crystal PMN-PT, PMN-PT/epoxy composites have the advantages of lower acoustic impedance and higher flexibility. In 2011, Zhou et al. fabricated a 64-element, radial pMUT array based on a PMN-PT/epoxy composite for endoscopic ultrasound imaging applications [102]. Starting from a 165 μm thick PMN-PT/epoxy 1-3 composite, followed by bonding impedance-matching and backing layers with an electrically conductive adhesive, cutting, and then wrapping on a cylinder, the radial pMUT array was fabricated with inner and outer diameters of 6 mm and 10 mm, respectively. Figure 10 shows the fabrication procedure and a photo of the PMN-PT/epoxy 1-3 composite-based radial pMUT array. Measurement of the fabricated pMUT showed an ultrahigh electromechanical coupling factor ($k_{t}=0.81$), a center frequency of 6.91 MHz, and a −6 dB bandwidth of 102% which was ~30% larger than those of PZT pMUTs. 

**Figure 11:**
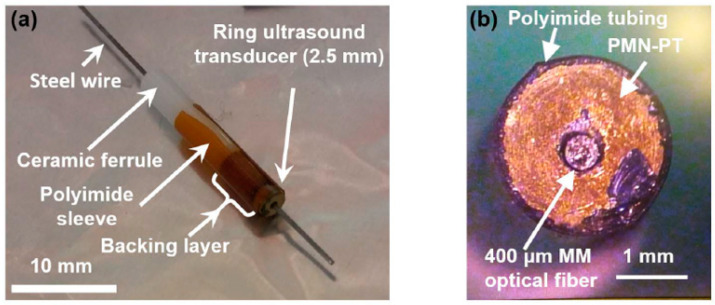
Optical image of the photoacoustic transducer device (**a**) and the front view of the pMUT surface (**b**) (Reproduced with permission from IEEE [103]).

PAI based on PMN-PT pMUTs has been demonstrated. For example, Dangi et al. reported a 2.5 mm diameter ring pMUT based on single-crystal PMN-PT with an optical fiber integrated for portable and wearable photoacoustic microscopy applications [103]. The fabricated pMUT had a center frequency of 17.25 MHz with a −6 dB bandwidth of 45%. An optical image of this photoacoustic transducer device is shown in Figure 11. This compact system shows the potential for endoscopic PAI applications. 

In 2012, Li et al. presented a 1.2 mm diameter catheter-based intravascular photoacoustic imaging probe based on PMN-PT pMUTs at 35 MHz and 80 MHz [71]. The low-frequency pMUT (35 MHz and 50% −6 dB bandwidth) was fabricated from a 55 μm thick PMN-PT crystal with an aperture size of 0.4 × 0.4 mm^2^, while the high-frequency pMUT (80 MHz and 45% −6 dB bandwidth) with the same aperture size was fabricated from a 30 μm thick PMN-PT free-standing film. Different probes were fabricated by incorporating either 35 MHz pMUT or 80 MHz pMUT. The spatial resolution and beam patterns of the probe were characterized by the intravascular imaging capability demonstrated in vitro in a rabbit aorta. The 35 MHz pMUT showed larger imaging depth while the 80 MHz pMUT demonstrated much finer resolution for delineating vessel boundaries. 

**Figure 12:**
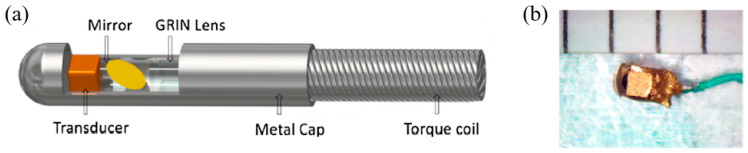
(**a**) Schematic of the imaging probe. (**b**) Photograph of the fabricated pMUT (Reproduced with permission from Elsevier [72]).

By dicing single-crystal PMN-PT and filling with epoxy, in 2019, Li et al. presented a 32 MHz PMN-PT/epoxy 1-3 composite-based pMUT for dual-modality photoacoustic and ultrasound endoscopy [72]. The fabricated pMUT, with an effective detection area of 0.5 × 0.5 mm^2^, was assembled into a miniature probe with an outer diameter of 1.45 mm (Figure 12). Phantom and in vivo animal studies were conducted, showing that this PMN-PT/epoxy 1-3 composite-based pMUT had enhanced the bandwidth (−6 dB bandwidth of 91.5%) and improved the signal-to-noise ratio compared with single-crystal PMN-PT and PZT ceramic-based pMUTs. 

##### Focused TEM pMUTs in Endoscopic PAI

To increase the imaging resolution, in addition to using superior piezoelectric materials, the focused-scanning scheme has been applied in endoscopic PAI, which usually focuses on both the optical excitation and acoustic detection [2]. Focused pMUTs based on different piezoelectric materials with spherical surfaces or focusing lenses have been applied in photoacoustic imaging endoscopes [40,61,62,64,70]. 

**Figure 13:**
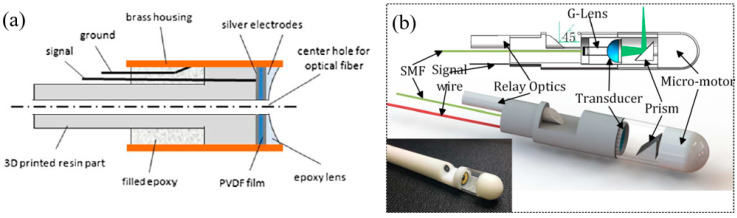
(**a**) Schematic of the lens-focused PVDF pMUT (Reproduced with permission from OSA [61]). (**b**) Schematic of the photoacoustic-hyperspectral imaging endoscope (Reproduced with permission from OSA [62]).

In 2016, Xiao et al. proposed a hollow-structured lens-focused PVDF pMUT for endoscopic PAI applications [61] (Figure 13a). The PVDF pMUT was fabricated based on a 52 μm thick PVDF film, which had a diameter of 6 mm and a center frequency of 9.2 MHz prior to attaching the acoustic lens. Experiments showed that a lateral resolution of less than 0.5 mm can be obtained with the focal length in the range of about 17–20 mm. In 2018, Liu et al. reported a photoacoustic and hyperspectral dual-modality endoscope with a diameter of 12 mm based on a 15 MHz spherically focused PVDF pMUT and demonstrated optical-resolution PAI of vascular structures and an oxygen saturation rate map in a rabbit’s rectum [62] (Figure 13b). Phantom experiments were also performed, showing the transverse resolution of 40 μm and the maximum imaging depth of 2 mm in the photoacoustic mode.

**Figure 14:**
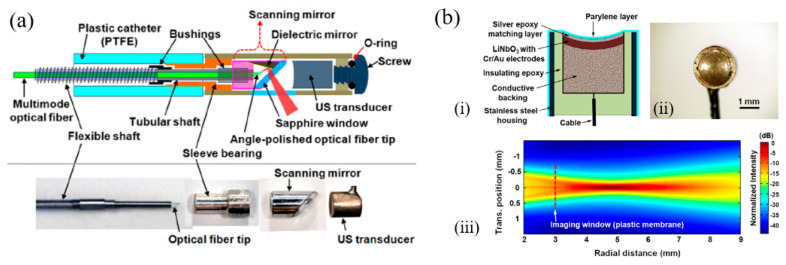
(**a**) Schematic (top) and photograph (bottom) of the distal section of the catheter-based photoacoustic endoscope. (**b**) Schematic (b-i), photograph (b-ii), and simulated transmission acoustic intensity map (b-iii) of the focused LiNbO_3_-based transducer (Reproduced with permission from SPIE [40]).

In 2014, Yang et al. developed a 3.2 mm photoacoustic endoscope based on a focused LiNbO_3_ pMUT that can be potentially used for imaging human gastrointestinal tracts [40] (Figure 14a). This endoscope consisted of a focused LiNbO_3_ pMUT (40 MHz, 1.4 mm aperture) with a focal length of 5 mm (Figure 14b). When an imaging target was placed at the focal point of the endoscope, photoacoustic B-scan images with a radial and transverse resolution of around 150 μm and 160 μm, respectively, were obtained. 

**Figure 15:**
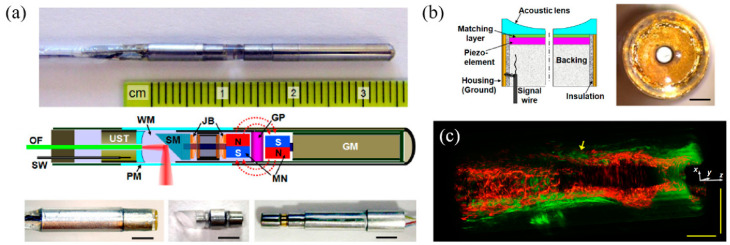
(**a**) Photograph (top) and schematic (middle) of the integrated photoacoustic/ultrasound imaging probe, and detailed photograph (bottom) of an optical fiber and ultrasound transducer unit, a scanning mirror unit, and a micromotor unit. (**b**) Schematic (left) and photograph (right) of the PMN-PT based transducer. (**c**) Three-dimensional (3D) rendered photoacoustic/ultrasound image of a rat colon (Reproduced with permission from OSA [70]). OF: optical fiber, SW: signal wire, UST: ultrasound transducer, WM: water medium, PM: plastic membrane, SM: scanning mirror, JB: jewel bearings, GP: glass partition, MN: magnets, GM: geared micromotor.

In 2012, Yang et al. developed a 2.5 mm diameter endoscopic probe for both photoacoustic and ultrasound imaging based on a focused PMN-PT pMUT [70]. The endoscope consists of three key components: an optical fiber and ultrasound transducer unit, a scanning mirror unit, and a micromotor unit, as shown in Figure 15a. The PMN-PT based ultrasound transducer has an outer diameter of ~1.8 mm and an inner hole diameter of ~0.5 mm. Acoustic focusing is achieved by a planoconcave plastic acoustic lens with a focal distance of ~4 mm (Figure 15b). The fabricated transducer has a center frequency of 33 MHz with a −6 dB fractional bandwidth of 60%. In vivo PAI was demonstrated in the colon of a rat, showing a radial and transverse resolution of ~58 μm and ~100 μm, respectively, when the target was placed at the focal position. Figure 15c shows a 3D image of a rat colon covering a length of 4 cm reconstructed by processing the data acquired during a scanning time of ~4 min at a 4 Hz frame rate. 

Although photoacoustic endoscopes based on focused pMUTs have demonstrated high-resolution PAI, the depth of focus (DOF) is sacrificed as a tradeoff. In addition to the constraints of DOF, the imaging speed is limited by the slow mechanical scanning of a single-element pMUT. This problem can be potentially overcome by using pMUT arrays with multichannel parallel data acquisition or phase-controlled imaging algorithms [104,105]. Yuan et al. presented a fast preclinical PAI endoscope based on a 64-element ring transducer array, which can receive photoacoustic signals in a 2π field of view without scanning [106]. The fabricated probe has a diameter of 30 mm and the ring transducer array has an inner and outer diameter of 5 mm and 10 mm, respectively. However, for this probe, the more the number of the transducer elements, the more complicated the data acquisition becomes and the more expensive the system is. Moreover, for thickness mode pMUTs, fabricating transducer arrays with small size is still a challenge, especially for high-frequency transducer arrays. Thus, applying them for endoscopic imaging applications is not so feasible. Mechanical dicing to separate array elements from piezoelectric plates and back-filling with polymer fillers is a typical technique to fabricate arrays with frequencies less than 20 MHz, but it is expensive and complicated [54]. Thereby, another type of pMUTs, based on the flexural vibration mode, is being extensively investigated for endoscopic imaging applications, which is the focus of Section 2.2. 

#### 2.1.3. Summary of TEM pMUTs for Endoscopic PAI Applications

Table 3 summarizes the key findings of various types of TEM pMUTs for endoscopic PAI applications. In comparison, TEM pMUTs based on PVDF have advantages of broad bandwidth, but also have limitations of large size. The low piezoelectric constant of PVDF makes it challenging to reduce the size while keeping a high sensitivity to detect weak photoacoustic signals. In contrast, TEM pMUTs based on LiNbO_3_, PZT, and PMN-PT exhibit narrower bandwidths but can be made into much smaller sizes. Among all the piezoelectric materials, PZT and PMN-PT are the most popular materials for fabricating small pMUTs. For fabrication, PVDF is easy to cut and can be bent or rolled due to its polymer nature, while LiNbO_3_, PZT, and PMN-PT are fragile and require dicing saws or lasers to cut. Lapping and polishing are also required to thin piezoelectric bulk materials for fabricating TEM pMUTs based on LiNbO_3_, PZT, and PMN-PT. 

### 2.2. Flexural Vibration Mode pMUTs

**Figure 16:**
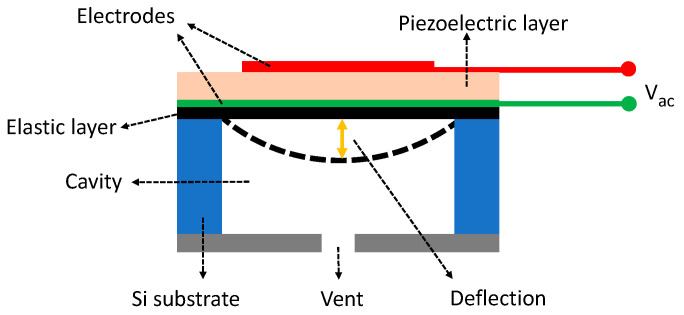
Typical structure of a flexural vibration mode pMUT in a cross-sectional view.

The structure of a typical flexural vibration mode (FVM) pMUT is shown in Figure 16 which consists of a thin multilayer membrane and an acoustic cavity. The membrane is composed of a piezoelectric active layer, two metal electrode layers, and an elastic layer. The elastic layer can be made of dielectrics such as SiO_2_ and Si_x_N_y_ or silicon. The pMUT works at the flexural vibration caused by the $d_{31}$ mode excitation of the piezoelectric membrane and can function as either an acoustic transmitter or an acoustic receiver. When an ultrasound wave comes, the membrane will deflect and vibrate due to the direct piezoelectric effect, thus generating an electrical signal. On the contrary, an ultrasound wave will be generated when an AC voltage is applied across the piezoelectric membrane due to the converse piezoelectric effect.

In the following, the materials, fabrication, and modeling of FVM pMUTs will be introduced first. Then, the applications of those FVM pMUTs in endoscopic PAI will be reviewed. Finally, the sensitivity and bandwidth enhancement will be discussed. 

#### 2.2.1. Materials and Fabrication

Thin-film deposition processes for several piezoelectric materials including ZnO, AlN, and PZT have been developed based on sputtering and sol–gel methods and widely applied in piezoelectric MEMS fabrication [107,108,109,110]. Among them, sputtered ZnO has been explored for fabricating pMUTs, but ZnO films have issues in biomedical applications due to its instability in aqueous solutions [111]. Moreover, ZnO films’ easy formation of oxygen vacancies, fast Zn diffusion, and being vulnerable to most acids greatly limit their applications [112,113]. In contrast, sputtered AlN, which has better chemical and thermal stability than ZnO, is increasingly attracting interest for the fabrication of pMUTs [114,115]. The small dielectric constant of AlN makes it a good candidate for sensing applications, but also makes it vulnerable to parasitic effects. On the other hand, sol–gel PZT and sputtered PZT, which have much higher piezoelectric constants and electromechanical coupling coefficients than ZnO and AlN, have been extensively used for fabricating pMUTs [116,117]. A comparison of the material properties of these commonly used piezoelectric thin films is shown in Table 4.

The key processes in thin-film pMUT fabrication include the deposition of the piezoelectric layer and the formation of the membrane since the frequency response of the pMUT is largely dependent on the piezoelectric properties and the dimensions of the membrane. AlN and PZT are dominant piezoelectric materials in pMUT fabrication. AlN films for pMUTs are typically deposited by sputtering with a thickness of 0.7–2 μm [124,125,126]. High residual stress and low deposition rate (less than 25 nm/min) are the limiting factors of sputtering AlN thin films [127]. PZT thin films used for pMUTs usually have a thickness of 0.6–2 μm, deposited by sol–gel or sputtering methods [123,128]. Thicker sol–gel PZT films require multiple coatings and high-temperature annealing, which will induce serious stress issues. Sputtering PZT is also very challenging to achieve high-quality PZT films with a thickness of over 2 μm. Limited thickness and much lower piezoelectric coefficient than bulk piezoelectric materials are still the drawbacks of AlN and PZT thin films. Additionally, the piezoelectric properties of deposited thin films are strongly dependent on the crystal orientation, which is related to the substrate and processing parameters. The deposition of high-quality piezoelectric films usually requires proper buffer layers, which can prevent interdiffusion and oxidation and help lower residual stresses to minimize the degradation of piezoelectric properties.

**Figure 17:**
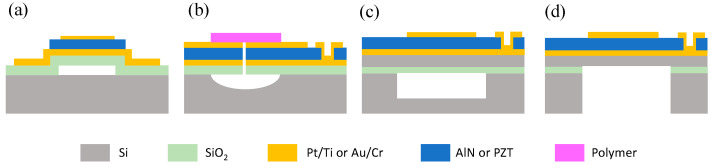
Cross-section schematic of pMUTs showing the membranes fabricated with (**a**) sacrificial layer releasing, (**b**) front-side etching, (**c**) cavity silicon-on-insulator (SOI) wafer bonding, and (**d**) backside deep reactive ion etching (DRIE).

Fabrication of the pMUT membrane is another important process. Currently, there are four kinds of processes commonly used to form the membrane: sacrificial releasing, front-side etching, cavity silicon-on-insulator (SOI) wafer bonding, and backside etching, as illustrated in Figure 17. In the first method, the sacrificial materials are formed below the membrane and then etched away through the releasing holes or tunnels after completing the fabrication of all layers of the membrane [129] (Figure 17a). The sacrificial releasing method can avoid the sidewall undercut of deep silicon anisotropic etching, so it makes densely packed pMUT arrays. Another approach to release the membrane is to etch the underlying silicon isotropically through etching holes from the front-side (Figure 17b). One advantage of this method is that cavities of relatively small diameters can be achieved easily. However, it is difficult to precisely define the dimension of the membrane, and an additional layer is needed to seal the etching holes after releasing the membrane [130]. The third method fabricates the cavity and the membrane on two substrates separately and then bonds them together [131] (Figure 17c). This method can avoid the membrane broken in the etching and releasing methods caused by the stress or surface tension. However, this process requires a high accuracy of lithography and alignment. Finally, the backside etching process is widely used to form the cavity through a deep reactive ion etching (DRIE) of the silicon substrate from the backside (Figure 17d). This method works well with SOI wafers, which can use the buried oxide layer as the etching stop [132].

#### 2.2.2. Modeling

Resonance frequency, bandwidth, and sensitivity are the key parameters of pMUTs for PAI applications. These parameters will determine the PAI resolution and depth. High resolution can be achieved with high ultrasound frequency, but high frequency will also have high acoustic attenuation and thus lead to a reduced imaging depth [7]. Broad bandwidth and high sensitivity are also desired to achieve high-resolution and large imaging depth. In this section, pMUT modeling is presented to analyze those key parameters and predict the frequency response of pMUTs. A pMUT can be modeled as a clamped vibration membrane with its fundamental resonance frequency governed by the membrane dimensions and material properties. For a circular pMUT membrane, which is the most common design, its fundamental resonance frequency in air can be calculated as [131]:

(17) $$\;f_{air}=\frac{1}{2\pi }\cdot \frac{3.2^{2}}{a^{2}}\sqrt{\frac{D}{\rho }}\;$$

where *a*, *D*, and *ρ* are the radius, equivalent flexural rigidity, and average area mass density of the membrane, respectively. 

Since pMUTs in endoscopic PAI applications typically work in a liquid environment usually made of water or mineral oil as the coupling medium, the effect of the radiation mass from the liquid must be considered. The reduced resonance frequency in liquid is expressed as [133]:

(18) $$f_{liquid}=f_{air}\cdot {\left(1+\frac{M_{a}}{M_{m}}\right)}^{-1/2}\;$$

where $f_{liquid}$, $M_{a}$, and $M_{m}$ are the resonance frequency in liquid, the added mass, and the mass of the membrane, respectively. For the vibration of a clamped circular membrane, $M_{a}$, is given by [134],

(19) $$M_{a}\approx 0.67\pi \rho_{liquid}a^{3}\;$$

where $\rho_{liquid}$ is the mass density of the liquid and a is the membrane’s radius.

**Figure 18:**
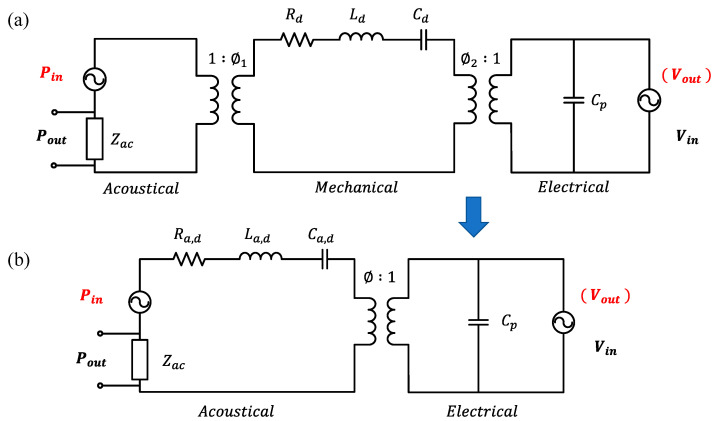
Lumped element modeling (LEM) circuit mode of a pMUT in three domains (**a**), and two domains (**b**).

Lumped element modeling (LEM) has been used to analyze the responses of pMUTs [133,135,136]. The LEM circuit model of FVM pMUTs is shown in Figure 18a, which represents a multiphysics system consisting of electrical, mechanical, and acoustical energy domains. In the mechanical domain, the effort represents the force (in N) and the flow represents the velocity of the membrane (in m/s). In the acoustical domain, the effort and flow correspond to the pressure (in Pa) and volume velocity (in m^3^/s), respectively. Since pMUTs can work as either transmitters or receivers, the LEM can represent either a voltage input with a pressure output or a pressure input with a voltage output.

The lumped elements in the LEM circuit model are defined as the following: $Z_{ac}$ is the acoustic radiation impedance of the membrane; $C_{d}$,$\;R_{d}$, and $L_{d}$ are the mechanical compliance, mechanical damping, and effective mass of the membrane; and $C_{p}$ is the electrical capacitance of the sandwiched piezoelectric element [133]. The acoustical and mechanical domains are coupled with the effective area of the membrane represented by $\emptyset_{1}$, while the mechanical and electrical domains are coupled by the piezoelectric effect represented by $\emptyset_{2}$. For a circular pMUT, $\emptyset_{1}$ is equal to one-third of the surface area of the membrane [133]. With $\emptyset_{1}$ known, the lumped elements in the mechanical domain can be transformed into the acoustical domain, so the LEM circuit in Figure 18a can be reduced to two domains, as shown in Figure 18b, which is convenient to derive the transfer function [136]. 

In PAI applications, pMUTs work only as acoustic receivers. By defining $Z_{m}$ as the overall impedance of the mechanoacoustic components in Figure 18b, the voltage generated by the pMUT in response to the received acoustic wave can be given as [136]:

(20) $$V_{out}=\frac{P_{in}\emptyset }{\emptyset^{2}+j\omega C_{p}Z_{m}}\approx \frac{P_{in}\emptyset }{j\omega C_{p}Z_{m}}\;$$

where $V_{out}$ is the voltage output and $P_{in}$ is the input pressure of the acoustic wave. Generally,$\;\omega C_{p}Z_{m}$ is much greater than $\emptyset^{2}$ for most pMUTs [136]. 

The quality factor Q can also be obtained by considering the energy dissipating components in the mechanoacoustic domain as:

(21) Q=1Ra,d+Re(Zac)La,dCa,d 

The resonance frequency $f_{0}$ and bandwidth Δf of a pMUT can also be calculated as:

(22) $$f_{0}=\frac{1}{2\pi }\sqrt{\frac{1}{L_{a,d}\;C_{a,d}}}\;$$

(23) Δf=f0Q=12π ·Ra,d+Re(Zac)La,d 

The modeling of pMUTs provides an efficient method to analyze the sensitivity, frequency response, and bandwidth of pMUTs. More details about pMUT design based on LEM are presented in [136,137]. 

#### 2.2.3. Advantages of Flexural Vibration Mode (FVM) pMUTs

Compared with thickness extension mode pMUTs, FVM pMUTs exhibit lower acoustic impedance and larger bandwidth. A FVM pMUT’s resonance frequency is not only dependent on the thickness of the piezoelectric layer but also determined by the membrane dimensions, the layer structures, and the material properties. Thus, there is much more design flexibility to choose the proper resonance frequency [138].

#### 2.2.4. FVM pMUTs for Endoscopic PAI Applications 

Due to their advantages discussed above, FVM pMUTs are drawing increasing attention in endoscopic PAI applications although it is still at an early stage. Primarily, sputtered AlN and sol–gel PZT have been used to fabricate FVM pMUTs for endoscopic PAI applications [124,139,140,141,142], which are reviewed below.

##### Sputtered AlN-Based FVM pMUTs 

**Figure 19:**
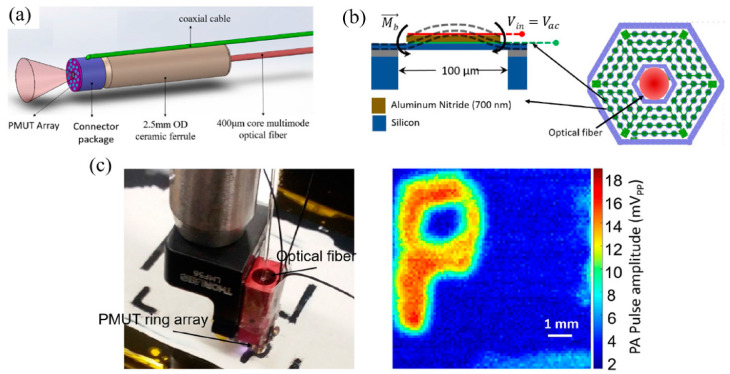
(**a**) Assembly drawing of the miniaturized PAI probe with the pMUT ring array and an optical fiber integrated. (**b**) Schematic of a pMUT cell and the ring pMUT array. (**c**) Photoacoustic imaging experimental setup (left) and the reconstructed image showing the predefined pattern ”P” (right) (Reproduced with permission from SPIE [124]).

In 2013, Chen et al. reported an AlN-based FVM pMUT with a resonance frequency of 2.885 MHz and obtained photoacoustic images of a human hair embedded in a tissue-mimicking phantom [139]. Though the sensitivity of this pMUT was relatively low, this work demonstrated the potential of using an AlN-based pMUT for PAI applications. In 2019, Dangi et al. developed a ring-shaped AlN-based FVM pMUT array specifically for endoscopic PAI applications [124]. As shown in Figure 19a, their pMUT ring array has an inner and outer diameter of 0.5 mm and 2.5 mm, respectively, which allows the insertion of an optical fiber for laser delivery. Each pMUT element has a membrane diameter of 100 μm and a resonance frequency of 6 MHz. The AlN film used in this work was deposited by DC magnetron sputtering with a thickness of 700 nm (Figure 19b). The wafer was annealed for 3 h at 800 °C after sputtering to reduce the residual stresses of the AlN film. PAI experiments were conducted using a black absorbing card as a sample; the sample was successfully imaged at 5 mm away from the pMUT array and shown in Figure 19c.

Although the potential of using AlN-based FVM pMUTs for endoscopic PAI applications has been demonstrated, their low sensitivities due to the low piezoelectric constant of AlN lead to small imaging depth. To overcome this issue, sol–gel and sputtered PZT thin films with higher piezoelectric constants have been explored, which is introduced below. 

##### Sol–Gel PZT-Based FVM pMUTs 

**Figure 20:**
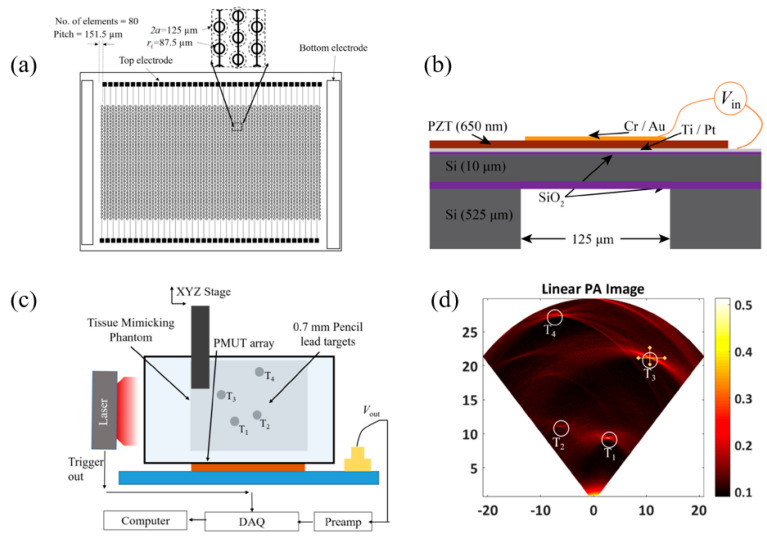
(**a**) Schematic of the 80-element linear pMUT array. (**b**) Cross-section schematic of the design of a single pMUT cell. (**c**) Experimental setup for PAI. (**d**) B-mode photoacoustic image showing 4 pencil lead targets embedded inside the agar phantom (Reproduced with permission from IEEE [141]).

In 2013, Liao et al. presented a 12 × 12 pMUT array based on a 0.6 μm sol–gel PZT with the membrane diameter and resonance frequency of each pMUT cell set at 50 μm and 10 MHz, respectively. [140]. In 2018, Dangi et al. presented an 80-element linear pMUT array based on a 0.65 μm sol–gel PZT for PAI applications [141] (Figure 20a). Each pMUT element contains 35 pMUT cells designed with a diameter of 125 μm and a center frequency of 7 MHz (Figure 20b). PAI was performed on an agar gel phantom with a pencil lead (0.7 mm diameter) embedded (Figure 20c). The pencil lead was successfully detected up to 2.3 cm deep, and B-mode imaging results showed that lateral and axial resolutions were ~600 μm and ~190 μm, respectively (Figure 20d). The frequency transform of the detected photoacoustic signals showed a center frequency of 7 MHz with a −6 dB fractional bandwidth of 68%. 

**Figure 21:**
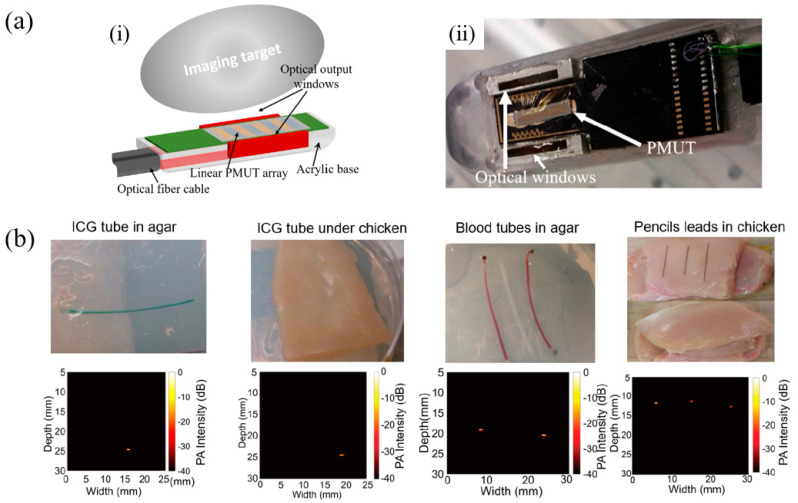
(**a**) Schematic representation (a-i) and photograph (a-ii) of the pMUT-based PAI probe. (**b**) Photographs (top) and corresponding B-mode photoacoustic images (bottom) of targets with different imaging conditions (Reproduced with permission from IEEE [142]).

In 2019, Dangi et al. reported a rectangular-shaped sol–gel PZT-based 65-element pMUT arrays [142]. The pMUT array was assembled into a fiber-optic PAI probe (Figure 21a). In this work, a 2% Nb-doped PZT film was used as the piezoelectric layer, which was formed by a sol–gel process that consisted of repeating the sol–gel deposition process 20 times to achieve a total thickness of 1.9 μm. PAI experiments were performed on various imaging targets embedded in agar gel phantoms or covered with chicken tissues (Figure 21b). An indocyanine green (ICG)-filled tube embedded in agar and covered with ~3 mm chicken tissue was clearly imaged at a ~25 mm distance from the pMUT-based PAI probe. Pencil leads (0.3 mm diameter) were also detected through an 8 mm thick chicken tissue. The frequency spectrum of the detected photoacoustic pulses showed that the fabricated pMUT had a center frequency of 6.75 MHz with a bandwidth of 89%. 

#### 2.2.5. Sensitivity and Bandwidth Enhancement of FVM pMUTs

Low sensitivity and insufficient bandwidth are the major challenges of FVM pMUTs for PAI. To further improve sensitivity, doping piezoelectric thin films to increase piezoelectric coefficients [143,144,145,146], directly using ceramic PZT with high piezoelectric coefficients [147,148], and special structure designs [149,150,151,152,153] have been proposed and investigated. Mixing multiple pMUT elements with different resonance frequencies into one array [146] and designing pMUT structures with multiple resonance modes [154,155,156] have also been explored to increase the bandwidth. 

##### Doping Piezoelectric Thin Films 

**Figure 22:**
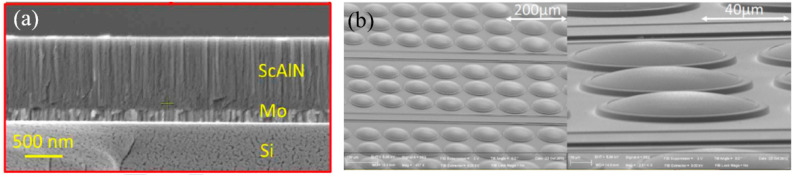
(**a**) SEM image of the sputtered Sc doped AlN film (Reproduced with permission from IEEE [144]). (**b**). SEM images of the Nb-doped PZT (PNZT)-based pMUT array showing dome-shaped elements with different dimensions (Reproduced with permission from AIP [146]).

Scandium (Sc) alloying has been studied as a means to increase the piezoelectric coefficient ($e_{31,f}$) of AlN [143]. In 2017, Wang et al. presented a pMUT based on a sputtered 1 μm thick scandium aluminum nitride (Sc_x_Al_1-x_N) (x = 15%) film [144] (Figure 22a). The presented Sc_x_Al_1-x_N pMUTs showed an increased transmission sensitivity by a factor of two compared to AlN-only pMUTs with the same dimensions, which is consistent with a 60% increase in $e_{31,f}$ by using 15% Sc. The electromechanical coupling coefficient ($k_{t}^{2}$) of ScAlN pMUTs was also 36% greater than the AlN-only pMUTs. In 2017, Zhou et al. developed a high signal-to-noise ratio (SNR) pMUT based on a *c*-axis-oriented Pb(Mn_1/3_, Nb_2/3_)O_3_-Pb(Zr, Ti)O_3_ (PMnN-PZT) epitaxial thin film with a thickness of 2 μm, showing a much higher sensitivity than polycrystalline PZT pMUTs owing to PMnN-PZT’s larger piezoelectric coefficient ($e_{31,f}=-14$ C/m^2^) and smaller dielectric constant ($\varepsilon_{r}=200-300$) [145]. Sputtered niobium (Nb)-doped PZT (PNZT) films also exhibit a higher piezoelectric coefficient ($e_{31,f}=-23$ C/m^2^) than sputtered PZT films. In 2012, Hajati et al. presented a three-dimensional pMUT array with dome-shaped elements based on a PNZT film with a high doping level of Nb (13%), showing high electromechanical coupling (45%) and improved acoustic sensitivity [146]. In this work, a bandwidth greater than 55% was also achieved by arranging dome-shaped pMUT elements with different dimensions into one array (Figure 22b). 

##### Thin Ceramic PZT

**Figure 23:**
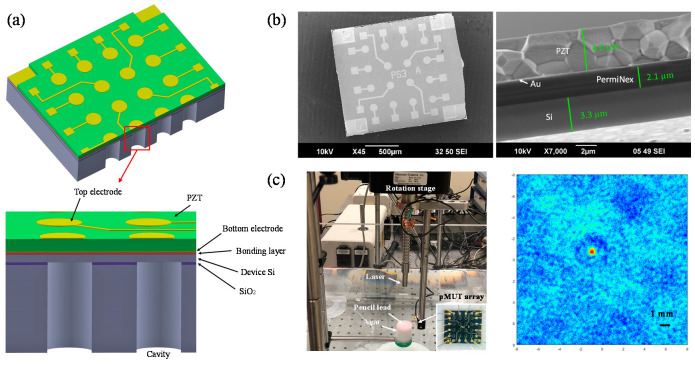
Ceramic PZT-based pMUT arrays for PAI. (**a**) Three-dimensional (3D) model of the pMUT array. (**b**) SEM images of the fabricated pMUT array (left) and its thin membrane in cross-section view (right). (**c**) Experimental setup (left) and reconstructed image (right) in PAI experiments (Reproduced with permission from IEEE [148]).

In addition to doping sputtered AlN or sputtered PZT thin films, in 2018, Wang et al. proposed to directly use bulk ceramic PZT for fabricating high-sensitivity FVM pMUTs [147]. By using wafer bonding and chemical mechanical polishing (CMP) techniques, In 2020, Wang et al. thinned down ceramic PZT to only 4 μm and fabricated ceramic PZT-based FVM pMUT arrays for endoscopic PAI [148] (Figure 23). Compared with sol–gel or sputtered PZT with a typical thickness of less than 2 μm, thinned ceramic PZT not only exhibits over four times greater piezoelectric constants, but also has no thickness limitation, thus enabling the fabrication of larger pMUTs to improve the sensitivity under the same frequency [148]. 

##### Special Structure Designs 

**Figure 24:**
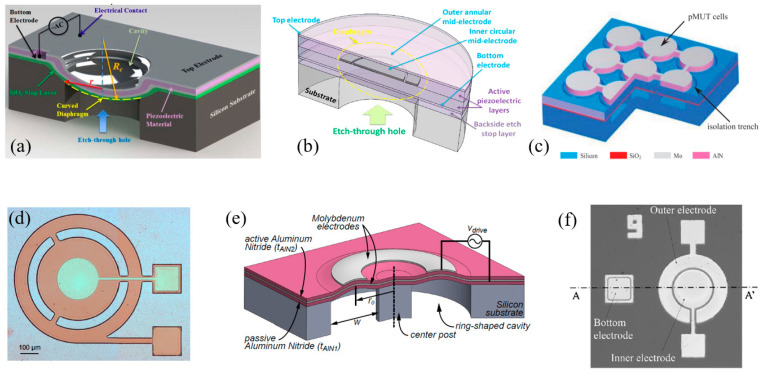
Schematic drawing or optical images of (**a**) curved diaphragm pMUT (Reproduced with permission from IEEE [126]), (**b**) bimorph pMUT (Reproduced with permission from IEEE [149]), (**c**) pMUT array with an isolation trench between cells (Reproduced with permission from IEEE [151]), (**d**) flexurally suspended pMUT (Reproduced with permission from IEEE [152]), (**e**) ring-shaped pMUT (Reproduced with permission from TRF [153]), and (**f**) dual-electrode pMUT (Reproduced with permission from Elsevier [145]).

In 2014, Akhbari et al. presented an AlN pMUT based on a curved diaphragm with a diameter of 140 μm and a radius of curvature of 1065 μm, showing a 50 times higher DC transmission sensitivity than that of a planar device with the same dimension [126] (Figure 24a). In 2015, the same group also proposed a bimorph AlN pMUT that had two 0.95 μm thick AlN layers sandwiched by three Mo electrodes, showing a four times higher central displacement sensitivity than that of a unimorph AlN pMUT under similar actuation conditions [149,150] (Figure 24b). In 2016, Wang et al. reported a pMUT array with an isolation trench between cells to enhance the transmission sensitivity, showing that the presence of the isolation trench can reduce the tensile stress on the edge of the membrane and thus improve the output pressure by about 76% without affecting other characteristics of the pMUTs significantly [151] (Figure 24c). Flexurally suspended membranes [152] (Figure 24d), ring-shaped membranes [153] (Figure 24e), and dual-electrode configurations [145] (Figure 24f) are also proved to be effective in enhancing the sensitivity of pMUTs. 

##### Multimode Designs 

**Figure 25:**
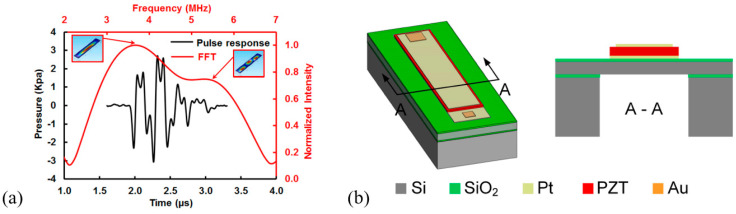
(**a**) Measured acoustic pressure of a broadband pMUT demonstrates a 97% fractional bandwidth with overlapping modes (Reproduced with permission from IEEE [155]). (**b**) Schematic design of a rectangular multifrequency pMUT (Reproduced with permission from IEEE [156]).

To enhance the bandwidth of pMUTs, in addition to mixing multiple pMUT elements with different dimensions into one array [146], designing multiple resonant modes in a specific frequency range is another effective method [154]. In 2015, Lu et al. demonstrated broadband PZT pMUTs with a 97% fractional bandwidth by utilizing a structure excited at two adjacent mechanical vibration modes [155] (Figure 25a). In 2018, Sun et al. investigated the difference and transition between the vibration modes of square and rectangular pMUTs, revealing that a pair of undegenerated string modes will occur in the desired frequency range when the pMUT diaphragm is designed as a rectangular shape with less symmetricity (Figure 25b). By designing rectangular pMUTs with optimized aspect ratios to gather more modes within specific frequency ranges, a −6 dB bandwidth of 112% was achieved [156]. 

#### 2.2.6. Summary of FVM pMUTs for Endoscopic PAI Applications

Table 5 summarizes the key findings of various types of FVM pMUTs for endoscopic PAI applications. Sputtered AlN and sol–gel PZT have been widely used as piezoelectric thin films for fabricating FVM pMUTs. Small single-element pMUTs and large arrays of pMUTs with various frequencies have been developed based on them. The typical thicknesses of sputtered AlN and sol–gel PZT are less than 2 μm. Sputtered PZT has a good combination of a high piezoelectric constant and stable physical properties but with limitations in high processing temperature and long process time. Up to this point, no reports of applying FVM pMUTs based on sputtered PZT for PAI applications have been found in the literature. In contrast, thin ceramic PZT obtained by CMP can have high piezoelectric constants, low-processing temperature, and a wide range of thicknesses. Ceramic PZT as thin as 4 μm has been achieved for fabricating FVM pMUTs. For fabrication, both sputtering and sol–gel processes require stress control of the deposited films. High cost is also a limitation of using sputtered AlN or PZT. In comparison, CMP ceramic PZT is a low-temperature process without stress concerns, but the precise thickness control of sub-10 μm is a challenge.

## 3. cMUTs

### 3.1. cMUT Basics

**Figure 26:**
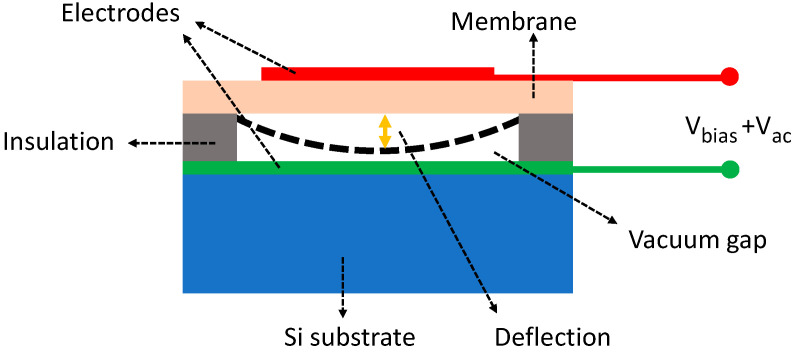
Typical structure of a capacitive MUT (cMUT) in cross-sectional view.

cMUTs are another type of MUTs whose development was started in the mid-1990s [157]. As shown in Figure 26, the basic structure of a cMUT is a capacitor consisting of a thin deformable membrane suspended over a shallow cavity (mostly a vacuum cavity). A metal layer on the top of the deformable membrane or the membrane itself, if conductive, forms the top electrode of the capacitor. A conductive layer on the substrate, separated by the shallow gap, acts as the bottom electrode that is usually used as the ground electrode. A cMUT can work as either an acoustic transmitter or an acoustic receiver. When the cMUT is used as a transmitter, an alternating voltage is applied to the top electrode so that an oscillating electrostatic force is induced and exerted on the deformable membrane that in turn vibrates and generates acoustic waves. When the cMUT is used as a receiver, a constant DC bias voltage is applied to the top electrode, so when an acoustic wave arrives, its acoustic pressure forces the deformable membrane to vibrate, causing electrical charges to flow in and out of the top electrode, i.e., generating an AC current with an amplitude related to the acoustic pressure amplitude and frequency, the capacitance and the bias voltage [158]. 

By using the lumped element method, a cMUT can be simplified as a parallel plate capacitor and modeled as a mass–spring–damper system, with a spring constant $k_{p}$, a mass $m_{p}$, and a damping constant $r_{p}$ (usually negligible). The acoustic impedance from the medium can be modeled by a damper $r_{m}$ and a mass $m_{m}$ [159]. With the lumped modeling, the resonance frequency of a cMUT is [159]:

(24) $$f_{0}=\frac{1}{2\pi }\sqrt{\frac{k_{p}-k_{s}}{m_{p}+m_{m}}}\;$$

where $k_{s}$ represents the spring softening effect generated by the DC bias, implying that the resonance frequency shifts as the DC bias voltage changes. 

The maximum receive sensitivity at its resonance frequency and the fractional bandwidth of a cMUT can be expressed as [159]:

(25) $$S_{R,max}={\left|\frac{I_{out}}{P_{in}}\right|}_{max}=\frac{E_{0}C_{0}A}{r_{m}}=\frac{V_{dc}}{g_{eff}-x_{dc}}\cdot \frac{C_{0}A}{r_{m}}$$

(26) $$\frac{\Delta f}{f_{0}}=\frac{r_{m}}{\sqrt{\left(m_{p}+m_{m}\right)\left(k_{p}-k_{s}\right)}}\;$$

where $P_{in}$ is the input pressure, $I_{out}$ is the output current, $E_{0}$ is the electric field, $C_{0}$ is the clamped capacitance of the cMUT at the bias voltage $V_{dc}$, *A* is the top electrode area of the cMUT, and $g_{eff}$ and $x_{dc}$ are the effective gap height under zero bias and gap change under the bias voltage $V_{dc}$, respectively. 

As indicated by Equation (25), the sensitivities of cMUTs are largely determined by how well the dimensions of the cavity can be controlled during the microfabrication and how large the DC bias can be applied. A small capacitive gap, large cMUT membrane, and high bias voltage will help improve the sensitivity, but there are tradeoffs in these parameters. A small capacitive gap will limit the displacement of the top electrode, thus restricting the maximum acoustic pressure level that can be generated. The dimension of a cMUT membrane will affect the equivalent mass and spring constant related to the resonance frequency as shown in Equation (24). Usually, cMUT membranes are in a circular shape and increasing the radius will reduce the resonance frequency. Considering the available microfabrication capability, the typical dimensions for cMUT cavities applied in PAI are listed in Table 6. The corresponding DC bias voltages, resonance frequencies, bandwidths, and NEPs are also summarized in Table 6 from the literature as an overview. 

As a newer generation of MEMS ultrasound transducers, cMUTs exhibit several advantages over conventional bulk piezoelectric transducers, including wide bandwidths, high electromechanical coupling coefficients, and easy fabrication for arrays with a broad range of frequencies and sizes [159]. High electromechanical coupling coefficients of over 0.8 have been demonstrated in cMUTs with the driving voltage close to the collapse voltage [171,172]. In this article, we will focus on the cMUTs that are specifically developed for PAI applications. 

### 3.2. cMUTs for PAI Applications

cMUTs have been researched for PAI applications with breakthroughs made in many aspects, such as two-dimensional (2D) cMUT arrays [161,162,163], transparent cMUTs [164,173,174], multiband cMUTs [165,166,167], and potential integration with endoscopes [168]. Most cMUTs have been studied and demonstrated on benchtop PAI systems. Applying cMUTs to endoscopic PAI is much more challenging, but there are a number of such attempts that have shown encouraging results. In the following, cMUTs in benchtop and endoscopic PAI systems will be reviewed, respectively. 

#### 3.2.1. cMUTs in Benchtop PAI Systems

Rectangular-shaped cMUT arrays: Two-dimensional (2D) cMUT arrays can avoid mechanically scanning single-element transducers by electronic scanning or beam-forming and thus greatly increase the imaging speed. Rectangular-shaped cMUTs have been developed and applied in benchtop PAI systems. For example, in 2009, Vaithilingam et al. developed 16 × 16 2D cMUT arrays for 3D PAI applications [162]. Each cMUT element had a membrane diameter of 30 μm and a center frequency of 3.48 MHz with a broad fractional bandwidth of 93.4%. The NEP was estimated to be 2.6 $\mathrm{mPa}/\sqrt{\mathrm{Hz}}$. Their PAI system successfully imaged black fishing lines of 180 μm in diameter. In 2012, Kothapalli et al. demonstrated deep-tissue PAI up to 5 cm using a 16 × 16 2D cMUT array (4 mm × 4 mm × 500 μm) with a center frequency of 5.5 MHz and a fractional bandwidth of 112% [163]. By having the preamp circuit flip-chip bonded on the cMUT array directly, the noise floor was reduced and a NEP of 1.1 $\mathrm{mPa}/\sqrt{\mathrm{Hz}}$ was achieved. PAI experiments showed that a horse black hair piece embedded at 5.3 cm depth in a chicken breast tissue was detected with a SNR of 23 dB. The calculated lateral and axial resolutions at 5.3 cm were 720 μm and 370 μm, respectively. 

**Figure 27:**
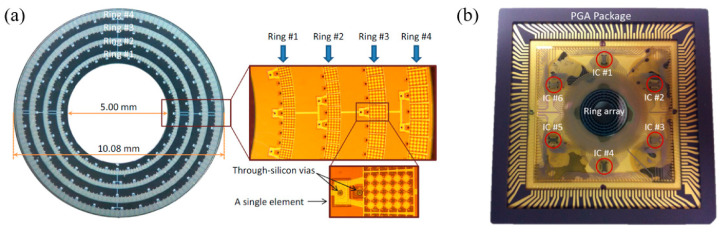
Optical pictures of the 512-element ring cMUT array (**a**) and the cMUT array integrated with custom front-end electronics in a PGA package (**b**) (Reproduced with permission from IEEE [168]).

Ring-shaped cMUT arrays: In addition to rectangular-shaped cMUT arrays, ring-shaped cMUT arrays were also developed [168]. Compared with rectangular-shaped array designs, ring-shaped arrays can produce lower side lobes because of their circular symmetry and spatial diversity. Ring-shaped cMUT arrays can also make better use of the space inside the circular outer tube of the PAI probe. Moreover, the central hole in the ring geometry can be utilized by optical fibers for light delivery in PAI. In 2013, Nikoozadeh et al. presented a ring-shaped cMUT array composed of 512 elements with an inner and outer diameter of 5.0 mm and 10.1 mm, respectively, for endoscopic ultrasound imaging and PAI [168]. As shown in Figure 27, the uniqueness of this cMUT array is that the cMUT elements were arranged into four concentric rings (diameters of numbers 1–4 are 6.0, 7.2, 8.5, and 9.7 mm, respectively) with each ring consisting of 128 elements and had different operational frequencies (frequencies of numbers 1–4 are 16, 12, 8, and 6.5 MHz, respectively) to achieve similar pressure beam profiles for all the rings. A broad bandwidth (−6 dB BW = 113%) was achieved. The proposed cMUT array shows great potential for endoscopic PAI applications.

**Figure 28:**
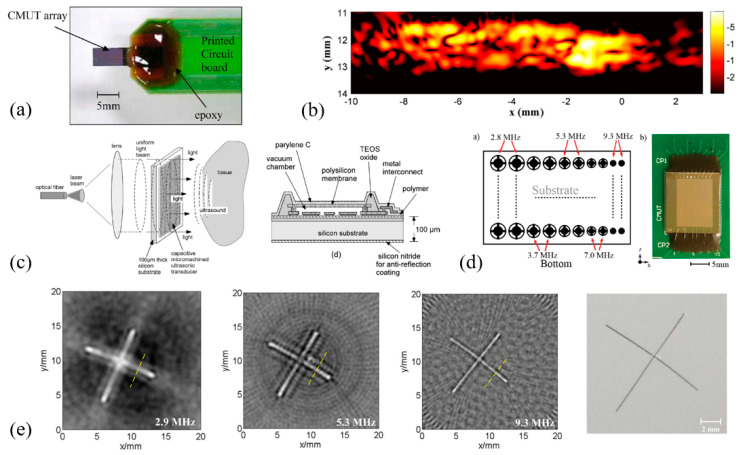
(**a**) Photograph of a 2D cMUT array wire bonded on a PCB (Reproduced with permission from IEEE [169]). (**b**) Reconstructed photoacoustic image of the lobster nerve cord (Reproduced with permission from IEEE [169]). (**c**) Schematic of a PAI system with backward illumination (left), and cross-section of the transparent cMUT used in this system (right) (Reproduced with permission from IEEE [173]). (**d**) Arrangement of cMUT elements with different frequency bands in an array (left), and a photograph of a multiband cMUT array bonded on a PCB (Reproduced with permission from IEEE [166]). (**e**). Photoacoustic images reconstructed from signals detected by cMUTs with different resonance frequencies, and a photograph of the phantom with two human hairs used in the experiment (Reproduced with permission from IEEE [166]).

Transparent cMUTs: Other than illuminating imaging targets in front of ultrasound transducers by integrating optical fibers with ring-shaped cMUT arrays or arranging the light source at a right angle to the acoustic path, back illumination has been explored by developing transparent cMUT arrays, which can avoid the shadowing problems associated with ultrasound transducers and facilitate the design of compact probes [164]. In 2010, Cheng et al. proposed a 2D cMUT array based on a thin silicon substrate which is relatively transparent to near-infrared (NIR) for minimally invasive PAI [169]. The fabricated 2D cMUT arrays were integrated on a 100 μm thick silicon bar (Figure 28a). Each circular cMUT element had a diameter of 46 μm, which showed a center frequency of 5 MHz with a −6 dB fractional bandwidth of 116%. Photoacoustic images of a lobster nerve cord were reconstructed using the developed cMUT array (Figure 28b). In 2012, Chen et al. further demonstrated PAI by launching a light source that transmits through the cMUT array to image the target of interest [173] (Figure 28c). By thinning the silicon substrate down to 100 μm and coating it with antireflection dielectric films, a light transmission rate of 12% was measured with the fabricated cMUT array in the NIR range of around 1.06 μm. 

Visible-light-transparent cMUTs were also developed [170,174]. By using a glass wafer as the substrate, a photo BCB polymer as the bonding agent, silicon nitride as the membrane material, and indium-tin-oxide (ITO) as the transparent electrodes, in 2019, Li et al. presented cMUTs with a transparency of up to 82% in the visible range for PAI applications [174]. With this method, in 2020, Ilkhechi et al. also developed transparent cMUT arrays for real-time PAI applications, showing an average transparency of 70% in the visible wavelength and up to 90% in the NIR range [170]. The reported NEP are 67 $\mathrm{mPa}/\sqrt{\mathrm{Hz}}$ and 10.4 $\mathrm{mPa}/\sqrt{\mathrm{Hz}}$ with the bias voltages of 40 V and 250 V, respectively. These transparent cMUTs generally have higher NEP, but they have great potential for further miniaturizing handheld and endoscopic PAI probes. 

Multiband cMUTs: Multiband cMUTs have been developed to detect wideband photoacoustic signals to provide both high-resolution images with high-frequency cMUTs and large imaging depth with low-frequency cMUTs. In 2018, Pun et al. designed a monolithic multiband cMUT array by integrating cMUT elements of various sizes with five different center frequencies (2.8, 3.7, 5.1, 7.3, and 9.4 MHz, respectively) into one array and demonstrated its photoacoustic imaging performances with human hairs embedded in a phantom, showing the high-frequency cMUTs can achieve high-resolution images while low-frequency cMUTs can present high signal-to-noise ratio (SNR) [166] (Figure 28d,e). 

#### 3.2.2. cMUTs in Endoscopic PAI Systems

**Figure 29:**
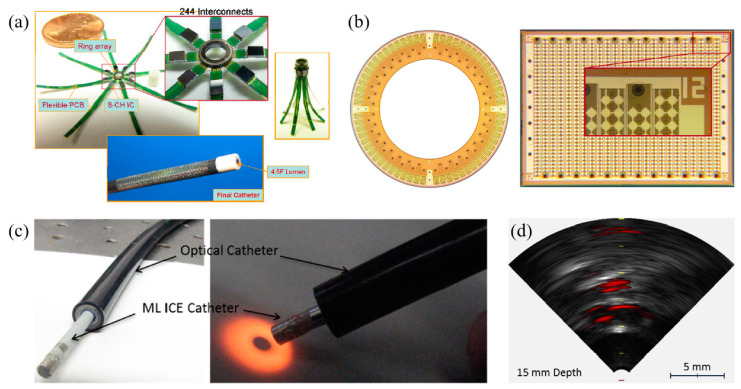
(**a**) Assembled ring cMUT array-based catheter (Reproduced with permission from IEEE [175]). (**b**) Optical pictures of ring and microlinear cMUT arrays (Reproduced with permission from IEEE [175]). (**c**) Assembled microlinear cMUT array-based photoacoustic catheter (Reproduced with permission from IEEE [176]). (**d**) Photoacoustic image of mouse subcutaneous kidney model (Reproduced with permission from IEEE [176]).

cMUT arrays have also been applied in endoscopic PAI systems. For example, in 2010, Nikoozadeh et al. reported both linear and ring-shape cMUT arrays for intracardiac imaging applications [175]. They developed a 24-element 1D cMUT phased-array and a 64-element ring cMUT array both with the center frequency of 10 MHz, and assembled them into a 3 mm diameter catheter and a 4 mm diameter catheter, respectively (Figure 29a). The 1D cMUT array had a dimension of 1.7 mm × 1.3 mm, while the ring cMUT array had an inner and outer diameter of 1.6 mm and 2.5 mm, respectively (Figure 29b). Custom designed integrated circuits (ICs) are required as a low-noise amplifier to improve the SNR and imaging quality because of the small capacitance (0.3 pF) of cMUT elements compared with the large parasitic capacitance (~200 pF) from microcoaxial cables. In 2012, Nikoozadeh et al. further demonstrated combined ultrasound and photoacoustic imaging based on the developed cMUT array-based 3 mm catheter [176]. This 3 mm cMUT catheter was assembled into a fiber-optical catheter with an outer diameter of 8 mm (Figure 29c). In their imaging experiments, the cMUT elements were biased at 50 V and excited with 55 V_pp_ AC pulses. Photoacoustic images of a mouse kidney tumor model were acquired, showing the vasculatures within the tumor (Figure 29d).

### 3.3. Limitations of cMUTs 

cMUT arrays have been developed and applied in endoscopic PAI applications due to their broad bandwidth and high sensitivity. However, cMUTs also have limitations in their fabrication and operation. Firstly, the small capacitive gap (typically in a range of 100–200 nm) of cMUTs is a fabrication challenge, which is a big concern for yield and requires complicated and precise fabrication process control. Secondly, due to their small active capacitance, cMUTs’ performance can be largely affected by the parasitics. Thirdly, a high bias voltage is required to achieve high sensitivity, which can cause safety concerns in biomedical in vivo imaging applications. Other reliability issues such as pull-in limitation, dielectric discharging and breakdown also constrain the applications of cMUTs.

## 4. Comparison of Photoacoustic Endoscopes Based on pMUTs and cMUTs

As reviewed in Section 2 and Section 3, several photoacoustic endoscopes have been developed based on pMUTs and cMUTs for different applications. Among them, pMUT-based photoacoustic endoscopes are more popular. In particular, thickness extension mode (TEM) pMUTs have advantages of high frequency and high sensitivity due to the superior piezoelectric constants of bulk piezoelectric materials such as single-crystal PMN-PT and ceramic PZT. Moreover, focused pMUT techniques can be applied to further improve sensitivity. Photoacoustic endoscopes based on TEM pMUTs have been extensively studied for a wide range of applications, especially for those high-resolution imaging applications such as visualization of blood vessel walls and detection of early-stage malignancies. However, small bandwidth and low imaging speed are the drawbacks of TEM pMUT-based photoacoustic endoscopes. Due to the acoustic impedance mismatch, most reported TEM pMUTs had a −6 dB fractional bandwidth of less than 60% even when impedance-matching layers are applied. Since it is expensive and complicated to fabricate TEM pMUT arrays, especially for high-frequency arrays, mechanical scanning is needed to acquire B-scan images for photoacoustic endoscopes based on single-element TEM pMUTs, thus limiting the frame rate of the imaging.

By contrast, flexural vibration mode (FVM) pMUTs are easy to be fabricated into arrays at low cost, which is advantageous to achieve high-speed imaging. FVM pMUTs also offer several advantages over cMUTs. First of all, FVM pMUTs do not require any air or vacuum gaps between the top and bottom electrodes, so the membrane deflection of FVM pMUTs is not limited as that of cMUTs is. Additionally, FVM pMUTs have a much larger capacitance and lower electrical impedance than cMUTs, making FVM pMUTs more robust against parasitic effects and better matched to the readout electronics [177]. Moreover, cMUTs need high bias voltage but FVM pMUTs do not need bias voltage at all. There is a significant interest in developing photoacoustic endoscopes based on FVM pMUTs although it is still at an early stage. Photoacoustic imaging has been demonstrated with the imaging targets covered by over 8 mm thick scattering tissues or placed over 25 mm away from the FVM pMUTs, showing the great potential of FVM pMUTs for achieving a large imaging depth in photoacoustic endoscopy. Although commonly used AlN and PZT thin films have much smaller piezoelectric constants than bulk PZT ceramics or single-crystal PMN-PT, leading to a reduced sensitivity of FVM pMUTs, various approaches have been proposed and verified to improve FVM pMUTs’ sensitivity, electromechanical coupling, and bandwidth. With more design flexibility than TEM pMUTs, FVM pMUTs have plenty of room for boosting their acoustic detection performance. 

In comparison, cMUT-based photoacoustic endoscopes have significant advantages in transducer bandwidth as most reported cMUTs can realize a broad −6 dB fractional bandwidth of over 100%, which is beneficial to achieve high axial imaging resolution. High-density 2D arrays, light-transparent designs, and easy integration of multiple frequency elements into an array are all the advantages of using cMUT-based photoacoustic endoscopes. However, a high bias voltage is always a concern of using cMUTs for endoscopic PAI. Moreover, reported cMUTs typically have center frequencies of less than 10 MHz with small membranes of around 30 μm in diameter. It is very challenging to fabricate high-frequency (over 40 MHz) cMUTs for high-resolution endoscopic PAI applications. High-frequency cMUTs operating at 45 MHz in immersion have been reported, but they only have a small membrane of 10 μm in diameter and a small capacitance of 0.12 pF [178]. Electrical impedance mismatching and vulnerability to parasitic capacitances are among the serious barriers that must be overcome for practical use. 

**Figure 30:**
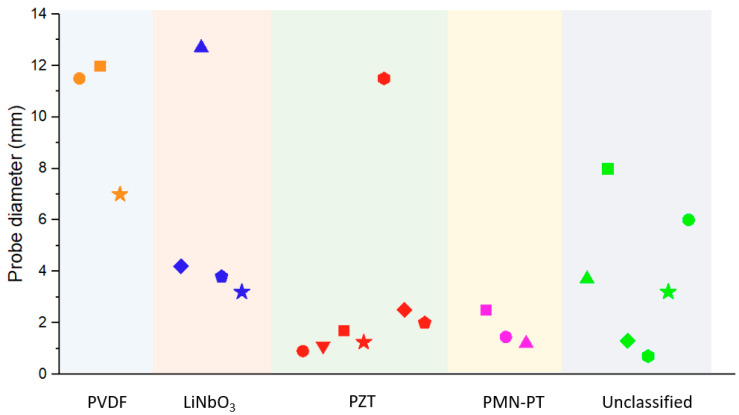
A comparison of the sizes of photoacoustic endoscopes based on TEM pMUTs (the markers are listed in Table 7).

Table 7 lists photoacoustic endoscopes based on single-element TEM pMUTs. Figure 30 is a scatter plot comparing their probe diameter versus piezoelectric material used. Compared with PVDF and LiNbO_3_, PZT and PMN-PT are more widely used for fabricating high-sensitivity TEM pMUTs that can fit into smaller endoscopic probes due to their higher piezoelectric coefficients. 

**Figure 31:**
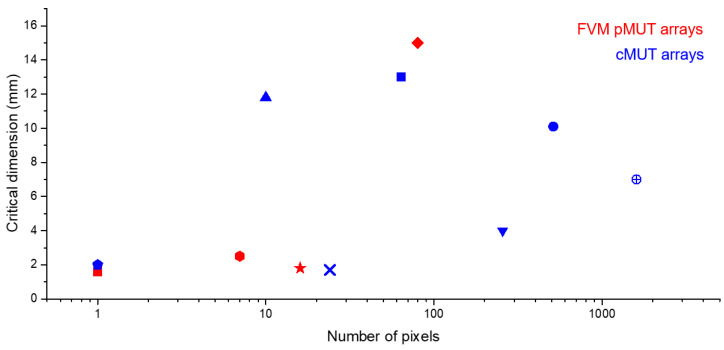
A comparison of FVM pMUT arrays and cMUT arrays for PAI applications (the markers are listed in Table 8).

Table 8 lists FVM pMUT arrays and cMUT arrays for PAI applications. Figure 31 is a scatter plot showing the critical dimension and number of pixels of each array. Unlike TEM pMUTs that are mostly single-element devices, FVM pMUT arrays and cMUT arrays have been developed with large numbers of pixels. Each pixel can also be composed of multiple pMUT or cMUT cells connected in parallel to increase the sensing area or bandwidth [140,141,164,170]. 

## 5. Summary and Outlook

In this article, we have reviewed MEMS ultrasound transducers, especially those for endoscopic PAI applications, summarized the progress of MEMS ultrasound transducers, including thickness extension mode pMUTs, flexural vibration mode pMUTs, and cMUTs, and discussed their respective advantages and limitations. MEMS ultrasound transducers with small size, high sensitivity, broad bandwidth, and low cost attributed to batch fabrication have greatly facilitated the development of photoacoustic imaging (PAI) endoscopes. In the last few years, several types of PAI endoscopes have been developed for various applications, such as intravascular imaging, human gastrointestinal tract imaging, and breast tumor margin evaluation and surgery guidance. Among the three types of MEMS ultrasound transducers used in these PAI endoscopes, each has its own advantages and limitations in achieving high-resolution, high-speed endoscopic PAI at large penetration depths. 

In the future, thickness extension mode pMUTs will be further studied with efforts to improve their bandwidth and develop new techniques to fabricate high-frequency transducer arrays at low cost. Flexural vibration mode pMUTs, with more design flexibility, will continue to be explored with better modeling and further enhancement of their sensitivity and bandwidth through innovations in terms of materials, fabrication techniques, and structure design optimization. cMUTs, with efforts in reducing bias voltage, improving electrical reliability, and high-level integration with electronic circuits, may find a broader application in endoscopic PAI. 

## Figures and Tables

**Table 1:** Comparison of piezoelectric materials applied in thickness extension mode piezoelectric micromachined ultrasound transducers (pMUTs) [54,55,56,57].

Property	PVDF	PMN-PT	LiNbO_3_ (36° Y-cut)	PZT-5H
Density (kg/m^3^)	1780	8060	4640	7500
Sound speed (m/s)	2200	4610	7340	4580
Acoustic impedance (Mrayl)	3.9	37.1	34.0	34.4
Piezoelectric constant $\left|d_{33}\right|$ (pC/N)	33	2820	18–39	593
Clamped relative permittivity $\varepsilon_{33,\;r}^{S}$	5–13	680–800	39	1470
Electromechanical coupling *k_t_*	0.12–0.15	0.58	0.49	0.51
Curie temperature (°C)	100	130	1150	200
Thickness for 5 MHz (µm)	220	461	734	458

**Table 2:** The noise equivalent pressure (NEP) and $p_{m}$ values of thickness extension mode pMUTs based on different piezoelectric materials (5 MHz center frequency, 2 MHz bandwidth).

Parameters	PVDF	PMN-PT	LiNbO_3_ (36° Y-cut)	PZT-5H
$p_{m}$ (Pa)	0.3	0.03	0.29–0.64	0.15
NEP ($\mathrm{mPa}/\sqrt{\mathrm{Hz}}$)	0.21	0.02	0.21–0.45	0.11

**Table 3:** Key findings of TEM pMUTs for endoscopic PAI applications.

Material	Frequency Range (MHz)	Bandwidth (−6 dB)	Piezoelectric Layer Thickness (μm)	Device Size (mm)	Fabrication
PVDF	2.5–15	84–150%	50–110	6–12	Easy cut
LiNbO_3_	13–50	60–75%	60–250	1.4–8	Lapping, dicing
PZT	5.5–40	30–65%	50–360	0.6–11.5	Lapping, dicing/laser cutting, dice-and-fill
PMN-PT	17–80	45–90%	30–110	0.5–2.5	Lapping, dicing/laser cutting, dice-and-fill

**Table 4:** Material properties of commonly used piezoelectric thin films [118,119,120,121,122,123].

Property		ZnO	AlN	Sol–gel PZT	Sputtered PZT
Density (kg/m^3^)		5700	3260	7700	7700
Young’s modulus (GPa)		98.6	283	96	96
Piezoelectric constants	$\left|d_{31}\right|$ (pC/N)	3.9–5.5	2–2.6	100–130	84–102
	$\left|e_{31,f}\right|$ (C/m^2^)	1.2	1.05	9.6–17.7	9–13
Dielectric constant $\varepsilon_{33,r}$		8.8	8.5–10.7	650–1470	400–980

**Table 5:** Key findings of flexural vibration mode (FVM) pMUTs for endoscopic PAI applications.

Material	Frequency Range (MHz)	Bandwidth (−6 dB)	Piezoelectric Layer Thickness (μm)	Device Size (mm)	Fabrication
Sputtered AlN	2.9–6	43–75%	0.7	2.5	High cost, stress control
Sol–gel PZT	6–10	68–89%	0.6–1.9	1.6–15	Stress control
Ceramic PZT	1.2	23%	4	1.8	Thickness control

**Table 6:** Overview of typical parameters of cMUTs applied in PAI [160,161,162,163,164,165,166,167,168,169,170].

Parameters	Values			Resonance Frequency (MHz)			Fractional Bandwidth			$\mathrm{NEP}\;(mPa/\sqrt{Hz})$
	min	Typical	max	min	Typical	max	min	Typical	max	Typical
Capacitive gap (μm)	0.03	0.1–0.2	0.4	1.5	3–6	16	30%	90–110%	130%	1.1–100
Membrane radius (μm)	10	15–20	40							
DC bias voltage (V)	20	40–60	250							

**Table 7:** Summary of photoacoustic endoscopes based on TEM pMUTs.

Marker	Piezoelectric Material	Probe Diameter (mm)	Frequency (MHz)	Ref.
●	PVDF	11.5	2.5	[60]
■	PVDF	12	15	[62]
★	PVDF	7	7.3	[61]
◆	LiNbO_3_	4.2	43	[63]
▲	LiNbO_3_	12.7	40	[65]
⬟	LiNbO_3_	3.8	36	[66]
★	LiNbO_3_	3.2	40	[40]
●	PZT	0.9	40	[41]
▼	PZT	1.1	40	[46]
■	PZT	1.7	40	[67]
★	PZT	1.25	30	[68]
⬢	PZT	11.5	5.5	[43]
◆	PZT	2.5	40	[24]
⬟	PZT	2	40	[69]
■	PMN-PT	2.5	40	[70]
●	PMN-PT	1.45	32	[72]
▲	PMN-PT	1.2	35, 80	[71]
▲	Unclassified	3.7	45	[179]
■	Unclassified	8	15	[47]
◆	Unclassified	1.3	6	[23]
⬢	Unclassified	0.7	50	[45]
★	Unclassified	3.2	39	[44]
●	Unclassified	6	10	[39]

**Table 8:** Summary of FVM pMUT arrays and cMUT arrays for PAI applications.

Marker	Type of MUTs	Chip Dimension (mm)	Frequency (MHz)	Ref.
◆	PZT-based pMUT	~15	7	[141]
⬢	AlN-based pMUT	2.5	6	[124]
■	PZT-based pMUT	1.6	10	[140]
★	PZT-based pMUT	1.8	1.2	[148]
▼	cMUT	4	5.5	[163]
●	cMUT	10.1	6.5, 8, 12, 16	[168]
✖	cMUT	1.7	6.6–8.6	[176]
⬟	cMUT	2	1.4	[164]
▲	cMUT	11.8	4, 10	[167]
■	cMUT	13	8	[170]
⨁	cMUT	7	3.7	[161]

## References

[1] Upputuri P.K., Pramanik M. (2016). Recent advances toward preclinical and clinical translation of photoacoustic tomography: A review. J. Biomed. Opt..

[2] Zhou Y., Yao J., Wang L.V. (2016). Tutorial on photoacoustic tomography. J. Biomed. Opt..

[3] Mallidi S., Luke G.P., Emelianov S. (2011). Photoacoustic imaging in cancer detection, diagnosis, and treatment guidance. Trends Biotechnol..

[4] Kherlopian A.R., Song T., Duan Q., Neimark M.A., Po M.J., Gohagan J.K., Laine A.F. (2008). A review of imaging techniques for systems biology. BMC Syst. Biol..

[5] Aldrich M.B., Marshall M.V., Sevick-Muraca E.M., Lanza G., Kotyk J., Culver J., Wang L.V., Uddin J., Crews B.C., Marnett L.J. (2012). Seeing it through: Translational validation of new medical imaging modalities. Biomed. Opt. Express.

[6] Aldrich J.E. (2007). Basic physics of ultrasound imaging. Crit. Care Med..

[7] Zackrisson S., Van De Ven S.M.W.Y., Gambhir S.S. (2014). Light in and sound out: Emerging translational strategies for photoacoustic imaging. Cancer Res..

[8] Wang L.V. (2009). Multiscale photoacoustic microscopy and computed tomography. Nat. Photonics.

[9] Xu M., Wang L.V. (2006). Photoacoustic imaging in biomedicine. Rev. Sci. Instrum..

[10] Wang X., Pang Y., Ku G., Xie X., Stoica G., Wang L.V. (2003). Noninvasive laser-induced photoacoustic tomography for structural and functional in vivo imaging of the brain. Nat. Biotechnol..

[11] Tang J., Dai X., Jiang H. (2016). Wearable scanning photoacoustic brain imaging in behaving rats. J. Biophotonics.

[12] Ermilov S.A., Khamapirad T., Conjusteau A., Leonard M.H., Lacewell R., Mehta K., Miller T., Oraevsky A.A. (2009). Laser optoacoustic imaging system for detection of breast cancer. J. Biomed. Opt..

[13] Manohar S., Vaartjes S.E., Van Hespen J.C.G., Klaase J.M., Van Den Engh F.M., Steenbergen W., Van Leeuwen T.G. (2007). Initial results of in vivo non-invasive cancer imaging in the human breast using near-infrared photoacoustics. Opt. Express.

[14] Hu S., Wang L.V. (2010). Photoacoustic imaging and characterization of the microvasculature. J. Biomed. Opt..

[15] Jeon S., Song H.B., Kim J., Lee B.J., Managuli R., Kim J.H., Kim J.H., Kim C. (2017). In vivo photoacoustic imaging of anterior ocular vasculature: A random sample consensus approach. Sci. Rep..

[16] Wray P., Lin L., Hu P. (2019). Photoacoustic computed tomography of human extremities. J. Biomed. Opt..

[17] Sun Y., Sobel E.S., Jiang H. (2011). First assessment of three-dimensional quantitative photoacoustic tomography for in vivo detection of osteoarthritis in the finger joints. Med. Phys..

[18] Xi L., Jiang H. (2015). High resolution three-dimensional photoacoustic imaging of human finger joints in vivo. Appl. Phys. Lett..

[19] Vogt W.C., Jia C., Wear K.A., Garra B.S., Pfefer J. (2015). Quantitative assessment of photoacoustic tomography systems integrating clinical ultrasound transducers using novel tissue-simulating phantoms. Photons Plus Ultrasound Imaging Sens. 2015.

[20] Mehrmohammadi M., Joon Yoon S., Yeager D., Emelianov S.Y. (2013). Photoacoustic imaging for cancer detection and staging. Curr. Mol. Imaging.

[21] Leng X., Chapman W., Rao B., Nandy S., Chen R., Rais R., Gonzalez I., Zhou Q., Chatterjee D., Mutch M. (2018). Feasibility of co-registered ultrasound and acoustic-resolution photoacoustic imaging of human colorectal cancer. Biomed. Opt. Express.

[22] Agarwal A., Huang S.W., O’Donnell M., Day K.C., Day M., Kotov N., Ashkenazi S. (2007). Targeted gold nanorod contrast agent for prostate cancer detection by photoacoustic imaging. J. Appl. Phys..

[23] Jin D., Yang F., Chen Z., Yang S., Xing D. (2017). Biomechanical and morphological multi-parameter photoacoustic endoscope for identification of early esophageal disease. Appl. Phys. Lett..

[24] Li Y., Lin R., Liu C., Chen J., Liu H., Zheng R., Gong X., Song L. (2018). In vivo photoacoustic/ultrasonic dual-modality endoscopy with a miniaturized full field-of-view catheter. J. Biophotonics.

[25] Schneider B.P., Miller K.D. (2005). Angiogenesis of breast cancer. J. Clin. Oncol..

[26] Oraevsky A.A., Andreev V.A., Karabutov A.A., Esenaliev R.O. (1999). Two-dimensional optoacoustic tomography: Transducer array and image reconstruction algorithm. Laser-Tissue Interact. X Photochem. Photothermal Photomech..

[27] Lin L., Hu P., Shi J., Appleton C.M., Maslov K., Li L., Zhang R., Wang L.V. (2018). Single-breath-hold photoacoustic computed tomography of the breast. Nat. Commun..

[28] Esenaliev R.O., Karabutov A.A., Oraevsky A.A. (1999). Sensitivity of laser opto-acoustic imaging in detection of small deeply embedded tumors. IEEE J. Sel. Top. Quantum Electron..

[29] Winkler A.M., Maslov K., Wang L.V. (2013). Noise-equivalent sensitivity of photoacoustics. J. Biomed. Opt..

[30] Wang L.V., Hu S. (2012). Photoacoustic Tomography: In vivo imaging from organelles to organs. Science (80-).

[31] Ku G., Wang X., Stoica G., Wang L.V. (2004). Multiple-bandwidth photoacoustic tomography. Phys. Med. Biol..

[32] Zhang Q., Liu Z., Carney P.R., Yuan Z., Chen H., Roper S.N., Jiang H. (2008). Non-invasive imaging of epileptic seizures in vivo using photoacoustic tomography. Phys. Med. Biol..

[33] Shung K.K., Zipparo M.J. (1996). Ultrasonic transducers and arrays. IEEE Eng. Med. Biol. Mag..

[34] Wong C.M., Chen Y., Luo H., Dai J., Lam K.H., Chan H.L. (2017). Development of a 20-MHz wide-bandwidth PMN-PT single crystal phased-array ultrasound transducer. Ultrasonics.

[35] Shung K.K., Cannata J.M., Zhou Q.F. (2007). Piezoelectric materials for high frequency medical imaging applications: A review. J. Electroceramics.

[36] Zhang H.F., Maslov K., Stoica G., Wang L.V. (2006). Functional photoacoustic microscopy for high-resolution and noninvasive in vivo imaging. Nat. Biotechnol..

[37] Maslov K., Zhang H.F., Hu S., Wang L.V. (2008). Optical-resolution photoacoustic microscopy for in vivo imaging of single capillaries. Opt. Lett..

[38] Lee C., Kim J.Y., Kim C. (2018). Recent progress on photoacoustic imaging enhanced with microelectromechanical systems (MEMS) technologies. Micromachines.

[39] Guo H., Song C., Xie H., Xi L. (2017). Photoacoustic endomicroscopy based on a MEMS scanning mirror. Opt. Lett..

[40] Yang J.-M., Li C., Chen R., Zhou Q., Shung K.K., Wang L.V. (2014). Catheter-based photoacoustic endoscope. J. Biomed. Opt..

[41] Li Y., Gong X., Liu C., Lin R., Hau W., Bai X., Song L. (2015). High-speed intravascular spectroscopic photoacoustic imaging at 1000 A-lines per second with a 0.9-mm diameter catheter. J. Biomed. Opt..

[42] Qu Y., Li C., Shi J. (2018). Transvaginal fast-scanning optical-resolution photoacoustic endoscopy. J. Biomed. Opt..

[43] Xi L., Grobmyer S.R., Wu L., Chen R., Zhou G., Gutwein L.G., Sun J., Liao W., Zhou Q., Xie H. (2012). Evaluation of breast tumor margins in vivo with intraoperative photoacoustic imaging. Opt. Express.

[44] Wei W., Li X., Zhou Q., Shung K.K., Chen Z. (2011). Integrated ultrasound and photoacoustic probe for co-registered intravascular imaging. J. Biomed. Opt..

[45] Lei P., Wen X., Wang L., Zhang P., Yang S. (2019). Ultrafine intravascular photoacoustic endoscope with a 0.7 mm diameter probe. Opt. Lett..

[46] Bai X., Gong X., Hau W., Lin R., Zheng J., Liu C., Zeng C., Zou X., Zheng H., Song L. (2014). Intravascular optical-resolution photoacoustic tomography with a 1.1 mm diameter catheter. PLoS ONE.

[47] Li X., Xiong K., Yang S. (2019). Large-depth-of-field optical-resolution colorectal photoacoustic endoscope. Appl. Phys. Lett..

[48] Jung J., Lee W., Kang W., Shin E., Ryu J., Choi H. (2017). Review of piezoelectric micromachined ultrasonic transducers and their applications. J. Micromech. Microeng..

[49] Vallet M., Varray F., Boutet J., Dinten J.-M., Caliano G., Savoia A.S., Vray D. (2017). Quantitative comparison of PZT and CMUT probes for photoacoustic imaging: Experimental validation. Photoacoustics.

[50] Jiang X., Al-Jumaily A.M. (2018). Ultrasound transducers for biomedical imaging and therapy. J. Eng. Sci. Med. Diagnostics Ther..

[51] Manwar R., Kratkiewicz K., Avanaki K. (2020). Overview of ultrasound detection technologies for photoacoustic imaging. Micromachines.

[52] Chan J., Zheng Z., Bell K., Le M., Reza P.H., Yeow J.T.W. (2019). Photoacoustic imaging with capacitive micromachined ultrasound transducers: Principles and developments. Sensors.

[53] Zhen Q., Piyawattanamatha W. (2017). New endoscopic imaging technology based on MEMS sensors and actuators. Micromachines.

[54] Zhou Q., Lau S., Wu D., Kirk Shung K. (2011). Piezoelectric films for high frequency ultrasonic transducers in biomedical applications. Prog. Mater. Sci..

[55] Cannata J.M., Ritter T.A., Chen W.H., Silverman R.H., Shung K.K. (2003). Design of efficient, broadband single-element (20-80 MHz) ultrasonic transducers for medical imaging applications. IEEE Trans. Ultrason. Ferroelectr. Freq. Control.

[56] Kotopoulis S., Wang H., Cochran S., Postema M. (2010). Lithium niobate ultrasound transducers for high-resolution focused ultrasound surgery. Proc. IEEE Ultrason. Symp..

[57] Morita T., Niino T., Asama H., Tashiro H. (2001). Fundamental study of a stacked lithium niobate transducer. Jpn. J. Appl. Phys. Part 1 Regul. Pap. Short Notes Rev. Pap..

[58] Rhyne T.L. (1998). Characterizing ultrasonic transducers using radiation efficiency and reception noise figure. IEEE Trans. Ultrason. Ferroelectr. Freq. Control.

[59] Shen Z., Lu J., Tan C.W., Miao J., Wang Z. (2013). d33 mode piezoelectric diaphragm based acoustic transducer with high sensitivity. Sensors Actuators A Phys..

[60] Xi L., Sun J., Zhu Y., Wu L., Xie H., Jiang H. (2010). Photoacoustic imaging based on MEMS mirror scanning. Opt. Express.

[61] Xiao J., Li Y., Jin W., Peng K., Zhu Z., Wang B. (2016). Photoacoustic endoscopy with hollow structured lens-focused polyvinylidine fluoride transducer. Appl. Opt..

[62] Liu N., Yang S., Xing D. (2018). Photoacoustic and hyperspectral dual-modality endoscope. Opt. Lett..

[63] Yang J.-M., Maslov K., Yang H.-C., Zhou Q., Shung K.K., Wang L.V. (2009). Photoacoustic endoscopy. Opt. Lett..

[64] Yang J., Li C., Chen R., Rao B., Yao J., Yeh C., Danielli A., Maslov K., Zhou Q., Shung K.K. (2015). Optical-resolution photoacoustic endomicroscopy in vivo. Biomed. Opt. Express.

[65] Li C., Yang J.-M., Chen R., Yeh C.-H., Zhu L., Maslov K., Zhou Q., Kirk Shung K., Wang L.V. (2014). Urogenital photoacoustic endoscope. Opt. Lett..

[66] Yang J.M., Favazza C., Chen R., Yao J., Cai X., Maslov K., Zhou Q., Shung K.K., Wang L.V. (2012). Simultaneous functional photoacoustic and ultrasonic endoscopy of internal organs in vivo. Nat. Med..

[67] Li G., Ye Z., Liang S., Chen S.L. (2020). Miniature probe for dual-modality photoacoustic microscopy and white-light microscopy for image guidance: A prototype toward an endoscope. J. Biophotonics.

[68] Jansen K., van der Steen A.F.W., van Beusekom H.M.M., Oosterhuis J.W., van Soest G. (2011). Intravascular photoacoustic imaging of human coronary atherosclerosis. Opt. Lett..

[69] Dai X., Xi L., Duan C., Yang H., Xie H., Jiang H. (2015). Miniature probe integrating optical-resolution photoacoustic microscopy, optical coherence tomography, and ultrasound imaging: Proof-of-concept. Opt. Lett..

[70] Yang J.-M., Chen R., Favazza C., Yao J., Li C., Hu Z., Zhou Q., Shung K.K., Wang L.V. (2012). A 2.5-mm diameter probe for photoacoustic and ultrasonic endoscopy. Opt. Express.

[71] Li X., Wei W., Zhou Q., Shung K.K., Chen Z. (2012). Intravascular photoacoustic imaging at 35 and 80 MHz. J. Biomed. Opt..

[72] Li Y., Lu G., Chen J.J., Jing J.C., Huo T., Chen R., Jiang L., Zhou Q., Chen Z. (2019). PMN-PT/Epoxy 1-3 composite based ultrasonic transducer for dual-modality photoacoustic and ultrasound endoscopy. Photoacoustics.

[73] Kim H.J., Lee H., Ziaie B. (2007). A wideband PVDF-on-silicon ultrasonic transducer array with microspheres embedded low melting temperature alloy backing. Biomed. Microdevices.

[74] Mo J.H., Robinson A.L., Fitting D.W., Terry F.L., Carson P.L. (1990). Micromachining for improvement of integrated ultrasonic transducer sensitivity. IEEE Trans. Electron Devices.

[75] Sleva M.Z., Briggs R.D., Hunt W.D. (1996). A micromachined poly(vinylidene fluoride-trifluoroethylene) transducer for pulse-echo ultrasound applications. IEEE Trans. Ultrason. Ferroelectr. Freq. Control.

[76] Fleischman A., Modi R., Nair A., Talman J., Lockwood G., Roy S. (2003). Miniature high frequency focused ultrasonic transducers for minimally invasive imaging procedures. Sensors Actuators A Phys..

[77] Jung M., Kim M.G., Lee J.H. (2009). Micromachined ultrasonic transducer using piezoelectric PVDF film to measure the mechanical properties of bio cells. Proc. IEEE Sens..

[78] Chandrana C., Talman J., Pan T., Roy S., Fleischman A. (2010). Design and analysis of MEMS based PVDF ultrasonic transducers for vascular imaging. Sensors.

[79] Huang N., He M., Shi H., Zhao Y., Lu M., Zou X., Yao L., Jiang H., Xi L. (2018). Curved-array-based multispectral photoacoustic imaging of human finger joints. IEEE Trans. Biomed. Eng..

[80] Zangabad R.P., Springeling G., Noothout E., Beurskens R., De Jong N., Van Der Steen A.F.W., Van Soest G., Daeichin V. (2018). A kerfless PVDF array for photoacoustic imaging. IEEE Int. Ultrason. Symp. IUS.

[81] Daeichin V., Chen C., Ding Q., Wu M., Beurskens R., Springeling G., Noothout E., Verweij M.D., van Dongen K.W.A., Bosch J.G. (2016). A broadband polyvinylidene difluoride-based hydrophone with integrated readout circuit for intravascular photoacoustic imaging. Ultrasound Med. Biol..

[82] Xi L., Li X., Jiang H. (2012). Variable-thickness multilayered polyvinylidene fluoride transducer with improved sensitivity and bandwidth for photoacoustic imaging. Appl. Phys. Lett..

[83] Snook K.A., Zhao J.Z., Alves C.H.F., Cannata J.M., Chen W.H., Meyer R.J., Ritter T.A., Shung K.K. (2002). Design, fabrication, and evaluation of high frequency, single-element transducers incorporating different materials. IEEE Trans. Ultrason. Ferroelectr. Freq. Control.

[84] Zhou Q., Cannata J.M., Guo H., Huang C., Marmarelis V.Z., Shung K.K. (2005). Half-thickness inversion layer high-frequency ultrasonic transducers using LiNbO 3 single crystal. IEEE Trans. Ultrason. Ferroelectr. Freq. Control.

[85] Chen J., Dai J.Y., Zhang C., Zhang Z.T., Feng G.P. (2012). Bandwidth improvement of LiNbO_3_ ultrasonic transducers by half-concaved inversion layer approach. Rev. Sci. Instrum..

[86] Dangi A., Agrawal S., Kothapalli S.-R. (2019). Lithium niobate-based transparent ultrasound transducers for photoacoustic imaging. Opt. Lett..

[87] Foster F.S., Ryan L.K., Turnbull D.H. (1991). Characterization of lead zirconate titanate ceramics for use in miniature high-frequency (20–80 MHz) transducers. IEEE Trans. Ultrason. Ferroelectr. Freq. Control.

[88] Goldberg R.L., Smith S.W. (1994). Multilayer piezoelectric ceramics for two-dimensional array transducers. IEEE Trans. Ultrason. Ferroelectr. Freq. Control.

[89] Dauchy F., Dorey R.A. (2007). Thickness mode high frequency MEMS piezoelectric micro ultrasound transducers. J. Electroceramics.

[90] Vos H.J., Frijlink M.A., Droog E., Goertz D.E., Blacquiere G., Gisolf A., De Jong N., Van Der Steern A.F.W. (2005). Transducer for harmonic intravascular ultrasound imaging. IEEE Trans. Ultrason. Ferroelectr. Freq. Control.

[91] Lukacs M., Yin J., Pang G., Garcia R., Cherin E., Williams R., Mehi J., Foster F.S. (2006). Performance and characterization of new micromachined high-frequency linear arrays. IEEE Trans. Ultrason. Ferroelectr. Freq. Control.

[92] Zhang Q.Q., Djuth F.T., Zhou Q.F., Hu C.H., Cha J.H., Shung K.K. (2006). High frequency broadband PZT thick film ultrasonic transducers for medical imaging applications. Ultrasonics.

[93] Zhou Q., Wu D., Liu C., Zhu B., Djuth F., Shung K.K. (2010). Micro-machined high-frequency (80 MHz) PZT thick film linear arrays. IEEE Trans. Ultrason. Ferroelectr. Freq. Control.

[94] Jiang X.J., Liu M.W., Shi F.F., Wang W., Wu X.M., Chen J.Y. (2019). A microscale linear phased-array ultrasonic transducer based on PZT ceramics. Sensors.

[95] Dangi A., Agrawal S., Lieberknecht J., Zhang J., Kothapalli S. Ring ultrasound transducer based miniaturized photoacoustic imaging system. Proceedings of the 2018 IEEE SENSORS, IEEE.

[96] Yang C., Jian X., Zhu X., Lv J., Jiao Y., Han Z., Stylogiannis A., Ntziachristos V., Sergiadis G., Cui Y. (2020). Sensitivity enhanced photoacoustic imaging using a high-frequency PZT transducer with an integrated front-end amplifier. Sensors.

[97] Park S.E., Shrout T.R. (1997). Characteristics of relaxor-based piezoelectric single crystals for ultrasonic transducers. IEEE Trans. Ultrason. Ferroelectr. Freq. Control.

[98] Jiang X., Yuan J.R., Cheng A., Snook K., Cao P.J., Rehrig P.W., Hackenberger W.S., Lavalelle G., Geng X., Shrout T.R. (2006). Microfabrication of piezoelectric composite ultrasound transducers (PC-MUT). Proc. IEEE Ultrason. Symp..

[99] Zhou Q., Xu X., Gottlieb E.J., Sun L., Cannata J.M., Ameri H., Humayun M.S., Han P., Shung K.K. (2007). PMN-PT single crystal, high-frequency ultrasonic needle transducers for pulsed-wave Doppler application. IEEE Trans. Ultrason. Ferroelectr. Freq. Control.

[100] Peng J., Lau S.T., Chao C., Dai J.Y., Chan H.L.W., Luo H.S., Zhu B.P., Zhou Q.F., Shung K.K. (2010). PMN-PT single crystal thick films on silicon substrate for high-frequency micromachined ultrasonic transducers. Appl. Phys. A Mater. Sci. Process..

[101] Fei C., Yang Y., Guo F., Lin P., Chen Q., Zhou Q., Sun L. (2018). PMN-PT single crystal ultrasonic transducer with half-concave geometric design for IVUS imaging. IEEE Trans. Biomed. Eng..

[102] Zhou D., Cheung K.F., Chen Y., Lau S.T., Zhou Q., Shung K.K., Luo H.S., Dai J., Chan H.L.W. (2011). Fabrication and performance of endoscopic ultrasound radial arrays based on PMN-PT single crystal/epoxy 1-3 composite. IEEE Trans. Ultrason. Ferroelectr. Freq. Control.

[103] Dangi A., Agrawal S., Datta G.R., Srinivasan V., Kothapalli S.R. (2020). Towards a low-cost and portable photoacoustic microscope for point-of-care and wearable applications. IEEE Sens. J..

[104] Yin B., Xing D., Wang Y., Zeng Y., Tan Y., Chen Q. (2004). Fast photoacoustic imaging system based on 320-element linear transducer array. Phys. Med. Biol..

[105] Yang D.W., Xing D., Yang S.H., Xiang L.Z. (2007). Fast full-view photoacoustic imaging by combined scanning with a linear transducer array. Opt. Express.

[106] Yuan Y., Yang S., Xing D. (2010). Preclinical photoacoustic imaging endoscope based on acousto-optic coaxial system using ring transducer array. Opt. Lett..

[107] Taeg Lim W., Hyo Lee C. (1999). Highly oriented ZnO thin films deposited on Ru/Si substrates. Thin Solid Films.

[108] Dubois M.A., Muralt P. (1999). Properties of aluminum nitride thin films for piezoelectric transducers and microwave filter applications. Appl. Phys. Lett..

[109] Kanno I. Piezoelectric PZT thin films: Deposition, evaluation and their applications. Proceedings of the 2019 20th International Conference on Solid-State Sensors, Actuators and Microsystems & Eurosensors XXXIII (TRANSDUCERS & EUROSENSORS XXXIII).

[110] Smith G.L., Pulskamp J.S., Sanchez L.M., Potrepka D.M., Proie R.M., Ivanov T.G., Rudy R.Q., Nothwang W.D., Bedair S.S., Meyer C.D. (2012). PZT-based piezoelectric MEMS technology. J. Am. Ceram. Soc..

[111] Kannan P.K., Saraswathi R., Rayappan J.B.B. (2010). A highly sensitive humidity sensor based on DC reactive magnetron sputtered zinc oxide thin film. Sensors Actuators A Phys..

[112] Li J., Ren W., Fan G., Wang C. (2017). Design and fabrication of piezoelectric micromachined ultrasound transducer (pMUT) with partially-etched ZnO film. Sensors.

[113] Ali W.R., Prasad M. (2020). Piezoelectric MEMS based acoustic sensors: A review. Sensors Actuators A Phys..

[114] Hou R., Hutson D., Kirk K.J., Qing Fu Y. (2012). AlN thin film transducers for high temperature non-destructive testing applications. J. Appl. Phys..

[115] Lu Y., Tang H., Fung S., Boser B.E., Horsley D.A. (2016). Pulse-echo ultrasound imaging using an AlN piezoelectric micromachined ultrasonic transducer array with transmit beam-forming. J. Microelectromech. Syst..

[116] Belgacem B., Calame F., Muralt P. (2007). Piezoelectric micromachined ultrasonic transducers with thick PZT sol gel films. J. Electroceramics.

[117] Thao P.N., Yoshida S., Tanaka S. (2018). Fabrication and characterization of PZT fibered-epitaxial thin film on Si for piezoelectric micromachined ultrasound transducer. Micromachines.

[118] Li J., Wang C., Ren W., Ma J. (2017). ZnO thin film piezoelectric micromachined microphone with symmetric composite vibrating diaphragm. Smart Mater. Struct..

[119] Muralt P. (2000). PZT thin films for microsensors and actuators: Where do we stand?. IEEE Trans. Ultrason. Ferroelectr. Freq. Control.

[120] Watanabe S., Fujiu T., Fujii T. (1995). Effect of poling on piezoelectric properties of lead zirconate titanate thin films formed by sputtering. Appl. Phys. Lett..

[121] Griffin B.A., Williams M.D., Coffman C.S., Sheplak M. (2011). Aluminum nitride ultrasonic air-coupled actuator. J. Microelectromech. Syst..

[122] Calame F., Muralt P. (2007). Growth and properties of gradient free sol-gel lead zirconate titanate thin films. Appl. Phys. Lett..

[123] Liu Z., Yoshida S., Horie T., Okamoto S., Takayama R., Tanaka S. Characterization of epitaxial-PZT/Si piezoelectric micromachined ultrasonic transducer (PMUT) and its phased array system. Proceedings of the 2019 20th International Conference on Solid-State Sensors, Actuators and Microsystems & Eurosensors XXXIII (TRANSDUCERS & EUROSENSORS XXXIII).

[124] Dangi A., Agrawal S., Tiwari S., Jadhav S., Cheng C., Datta G.R., Troiler-McKinstry S., Pratap R., Kothapalli S.-R. Ring PMUT array based miniaturized photoacoustic endoscopy device. Proceedings of the Photons Plus Ultrasound: Imaging and Sensing 2019.

[125] Wang T., Lee C. (2015). Zero-bending piezoelectric micromachined ultrasonic transducer (pMUT) with enhanced transmitting performance. J. Microelectromech. Syst..

[126] Akhbari S., Sammoura F., Shelton S., Yang C., Horsley D., Lin L. Highly responsive curved aluminum nitride PMUT. Proceedings of the IEEE International Conference on Micro Electro Mechanical Systems.

[127] Ababneh A., Schmid U., Hernando J., Sánchez-rojas J.L., Seidel H. (2010). The influence of sputter deposition parameters on piezoelectric and mechanical properties of AlN thin films. Mater. Sci. Eng. B.

[128] Ling J., Chen Y.Q., Chen Y., Wang D.Y., Zhao Y.F., Pang Y., Yang Y., Ren T.L. (2018). Design and Characterization of high-density ultrasonic transducer array. IEEE Sens. J..

[129] Luo G.L., Fung S., Wang Q., Kusano Y., Lasiter J., Kidwell D., Horsley D.A. High fill factor piezoelectric micromachined ultrasonic transducers on transparent substrates. Proceedings of the 2017 19th International Conference on Solid-State Sensors, Actuators, and Microsystems.

[130] Griggio F., Demore C.E.M., Kim H., Gigliotti J., Qiu Y., Jackson T.N., Choi K., Tutwiler R.L., Cochran S., Trolier-Mckinstry S. Micromachined diaphragm transducers for miniaturised ultrasound arrays. Proceedings of the IEEE International Ultrasonics Symposium, IUS, IEEE.

[131] Lu Y., Horsley D.A. (2015). Modeling, fabrication, and characterization of piezoelectric micromachined ultrasonic transducer arrays based on cavity SOI wafers. J. Microelectromech. Syst..

[132] Dausch D.E., Castellucci J.B., Chou D.R., Von Ramm O.T. (2008). Theory and operation of 2-D array piezoelectric micromachined ultrasound transducers. IEEE Trans. Ultrason. Ferroelectr. Freq. Control.

[133] Lu Y., Heidari A., Horsley D.A. (2015). A high fill-factor annular array of high frequency piezoelectric micromachined ultrasonic transducers. J. Microelectromech. Syst..

[134] Blevins R.D. (1979). Formulas for Natural Frequency and Mode Shape.

[135] Przybyla R.J., Shelton S.E., Guedes A., Izyumin I.I., Kline M.H., Horsley D.A., Boser B.E. (2011). In-air rangefinding with an AlN piezoelectric micromachined ultrasound transducer. IEEE Sens. J..

[136] Dangi A., Pratap R. (2017). System level modeling and design maps of PMUTs with residual stresses. Sensors Actuators A Phys..

[137] Smyth K., Kim S.-G. (2015). Analytical equivalent circuit model for piezoelectric micromachined ultrasonic transducers. IEEE Trans. Ultrason. Ferroelectr. Freq. Control.

[138] Akasheh F., Myers T., Fraser J.D., Bose S., Bandyopadhyay A. (2004). Development of piezoelectric micromachined ultrasonic transducers. Sensors Actuators A Phys..

[139] Chen B., Chu F., Liu X., Li Y., Rong J., Jiang H. (2013). AlN-based piezoelectric micromachined ultrasonic transducer for photoacoustic imaging. Appl. Phys. Lett..

[140] Liao W., Liu W., Rogers J.E., Usmani F., Tang Y., Wang B., Jiang H., Xie H. Piezeoelectric micromachined ultrasound tranducer array for photoacoustic imaging. Proceedings of the 2013 17th International Conference on Solid-State Sensors, Actuators and Microsystems (TRANSDUCERS & EUROSENSORS XXVII).

[141] Dangi A., Agrawal S., Tiwari S., Jadhav S., Cheng C., Trolier-mckinstry S. Evaluation of high frequency piezoelectric micromachined ultrasound transducers for photoacoustic imaging. Proceedings of the IEEE Sensors.

[142] Dangi A., Cheng C.Y., Agrawal S., Tiwari S., Datta G.R., Benoit R.R., Pratap R., Trolier-Mckinstry S., Kothapalli S.R. (2020). A photoacoustic imaging device using piezoelectric micromachined ultrasound transducers (PMUTs). IEEE Trans. Ultrason. Ferroelectr. Freq. Control.

[143] Matloub R., Hadad M., Mazzalai A., Chidambaram N., Moulard G., Sandu C.S., . Metzger T., Muralt P. (2013). Piezoelectric Al_1-x_Sc_x_N thin films: A semiconductor compatible solution for mechanical energy harvesting and sensors. Appl. Phys. Lett..

[144] Wang Q., Lu Y., Mishin S., Oshmyansky Y., Horsley D.A. (2017). Design, fabrication, and characterization of scandium aluminum nitride-based piezoelectric micromachined ultrasonic transducers. J. Microelectromech. Syst..

[145] Zhou Z., Yoshida S., Tanaka S. (2017). Epitaxial PMnN-PZT/Si MEMS ultrasonic rangefinder with 2 m range at 1 V drive. Sensors Actuators A Phys..

[146] Hajati A., Latev D., Gardner D., Hajati A., Imai D., Torrey M., Schoeppler M. (2012). Three-dimensional micro electromechanical system piezoelectric ultrasound transducer. Appl. Phys. Lett..

[147] Wang H., Yu Y., Chen Z., Yang H., Jiang H., Xie H. Design and fabrication of a piezoelectric micromachined ultrasonic transducer array based on ceramic PZT. Proceedings of the IEEE Sensors.

[148] Wang H., Chen Z., Yang H., Jiang H., Xie H. (2020). A ceramic PZT-based pMUT array for endoscopic photoacoustic imaging. J. Microelectromech. Syst..

[149] Akhbari S., Sammoura F., Yang C., Mahmoud M., Aqab N., Lin L. Bimorph pMUT with dual electrodes. Proceedings of the IEEE International Conference on Micro Electro Mechanical Systems.

[150] Akhbari S., Sammoura F., Eovino B., Yang C., Lin L. (2016). Bimorph piezoelectric micromachined ultrasonic transducers. J. Microelectromech. Syst..

[151] Wang M., Zhou Y., Randles A. (2016). Enhancement of the transmission of piezoelectric micromachined ultrasonic transducer with an isolation trench. J. Microelectromech. Syst..

[152] Guedes A., Shelton S., Przybyla R., Izyumin I., Boser B., Horsley D.A. Aluminum nitride pMUT based on a flexurally-suspended membrane. Proceedings of the 2011 16th International Conference on Solid-State Sensors, Actuators, and Microsystems.

[153] Eovino B.E., Akhbari S., Lin L. Ring-shaped piezoelectric micromachined ultrasonic transducers (pMUT) with increased pressure generation. Proceedings of the Solid-State Sensors, Actuators Microsystems Workshop.

[154] Wang T., Kobayashi T., Lee C. Broadband piezoelectric micromachined ultrasonic transducer (pMUT) using mode-merged design. Proceedings of the 10th IEEE International Conference on Nano/Micro Engineered and Molecular Systems.

[155] Lu Y., Rozen O., Tang H.Y., Smith G.L., Fung S., Boser B.E., Polcawich R.G., Horsley D.A. Broadband piezoelectric micromachined ultrasonic transducers based on dual resonance modes. Proceedings of the IEEE International Conference on Micro Electro Mechanical Systems (MEMS).

[156] Sun C., Shi Q., Yazici M.S., Kobayashi T., Liu Y., Lee C. (2019). Investigation of broadband characteristics of multi-frequency piezoelectric micromachined ultrasonic transducer (MF-pMUT). IEEE Sens. J..

[157] Haller M.I., Khuri-Yakub B.T. (1996). A surface micromachined electrostatic ultrasonic air transducer. IEEE Trans. Ultrason. Ferroelectr. Freq. Control.

[158] Khuri-Yakub B.T., Oralkan Ö. (2011). Capacitive micromachined ultrasonic transducers for medical imaging and therapy. J. Micromech. Microeng..

[159] Brenner K., Ergun A.S., Firouzi K., Rasmussen M.F., Stedman Q., Khuri-Yakub B. (2019). Advances in capacitive micromachined ultrasonic transducers. Micromachines.

[160] Takezaki T., Kawano M., Hasegawa H., Machida S., Ryuzaki D. (2017). Ultra-narrow gap CMUT cell structure for highly sensitive photoacoustic imaging. IEEE Int. Ultrason. Symp. IUS.

[161] Chee R., Sampaleanu A., Rishi D., Zemp R. (2014). Top orthogonal to bottom electrode (TOBE) 2-D CMUT arrays for 3-D photoacoustic imaging. IEEE Trans. Ultrason. Ferroelectr. Freq. Control.

[162] Vaithilingam S., Ma T.J., Furukawa Y., Wygant I.O., Zhuang X., De La Zerda A., Oralkan Ö., Kamaya A., Gambhir S.S., Jeffrey R.B. (2009). Three-dimensional photoacoustic imaging using a two-dimensional CMUT array. IEEE Trans. Ultrason. Ferroelectr. Freq. Control.

[163] Kothapalli S.R., Ma T.J., Vaithilingam S., Oralkan Ö., Khuri-Yakub B.T., Gambhir S.S. (2012). Deep tissue photoacoustic imaging using a miniaturized 2-D capacitive micromachined ultrasonic transducer array. IEEE Trans. Biomed. Eng..

[164] Zhang X., Wu X., Adelegan O.J., Yamaner F.Y., Oralkan O. (2018). Backward-mode photoacoustic imaging using illumination through a CMUT with improved transparency. IEEE Trans. Ultrason. Ferroelectr. Freq. Control.

[165] Chee R.K.W., Zhang P., Maadi M., Zemp R.J. (2017). Multifrequency interlaced CMUTs for photoacoustic imaging. IEEE Trans. Ultrason. Ferroelectr. Freq. Control.

[166] Pun S.H., Yu Y., Zhang J., Wang J., Cheng C.H., Lei K.F., Yuan Z., Mak P.U. (2018). Monolithic multiband CMUTs for photoacoustic computed tomography with in vivo biological tissue imaging. IEEE Trans. Ultrason. Ferroelectr. Freq. Control.

[167] Zhang J., Pun S.H., Yu Y., Gao D., Wang J., Mak P.U., Lei K.F., Cheng C.-H., Yuan Z. (2017). Development of a multi-band photoacoustic tomography imaging system based on a capacitive micromachined ultrasonic transducer array. Appl. Opt..

[168] Nikoozadeh A., Chang C., Choe J.W., Bhuyan A., Lee B.C., Moini A., Khuri-Yakub P.T. An integrated ring CMUT array for endoscopic ultrasound and photoacoustic imaging. Proceedings of the IEEE International Ultrasonics Symposium, IUS, IEEE.

[169] Cheng X., Chen J., Li C. (2010). A miniature capacitive micromachined ultrasonic transducer array for minimally invasive photoacoustic imaging. J. Microelectromech. Syst..

[170] Ilkhechi A.K., Ceroici C., Li Z., Zemp R. (2020). Transparent capacitive micromachined ultrasonic transducer (CMUT) arrays for real-time photoacoustic applications. Opt. Express.

[171] Yaralioglu G.G., Ergun A.S., Bayram B., Hæggström E., Khuri-Yakub B.T. (2003). Calculation and measurement of electromechanical coupling coefficient of capacitive micromachined ultrasonic transducers. IEEE Trans. Ultrason. Ferroelectr. Freq. Control.

[172] Guldiken R.O., Zahorian J., Yamaner F.Y., Degertekin F.L. (2009). Dual-electrode CMUT with non-uniform membranes for high electromechanical coupling coefficient and high bandwidth operation. IEEE Trans. Ultrason. Ferroelectr. Freq. Control.

[173] Chen J., Wang M., Cheng J.C., Wang Y.H., Li P.C., Cheng X. (2012). A photoacoustic imager with light illumination through an infrared-transparent silicon CMUT array. IEEE Trans. Ultrason. Ferroelectr. Freq. Control.

[174] Li Z., Ilkhechi A.K., Zemp R. (2019). Transparent capacitive micromachined ultrasonic transducers (CMUTs) for photoacoustic applications. Opt. Express.

[175] Nikoozadeh A., Oralkan Ö., Gencel M., Choe J.W., Stephens D.N., De La Rama A., Chen P., Lin F., Dentinger A., Wildes D. (2010). Forward-looking intracardiac imaging catheters using fully integrated CMUT arrays. Proc. IEEE Ultrason. Symp..

[176] Nikoozadeh A., Choe J.W., Kothapalli S.R., Moini A., Sanjani S.S., Kamaya A., Oralkan O., Gambhir S.S., Khuri-Yakub P.T. Photoacoustic imaging using a 9F microLinear CMUT ICE catheter. Proceedings of the IEEE International Ultrasonics Symposium, IUS.

[177] Qiu Y., Gigliotti J.V., Wallace M., Griggio F., Demore C.E.M., Cochran S., Trolier-McKinstry S. (2015). Piezoelectric micromachined ultrasound transducer (PMUT) arrays for integrated sensing, actuation and imaging. Sensors.

[178] Oralkan Ö., Hansen S.T., Bayram B., Yarahoǧlu G.G., Ergun A.S., Khuri-Yakub B.T. (2004). High-frequency CMUT arrays for high-resolution medical imaging. Proc. IEEE Ultrason. Symp..

[179] Guo Z., Li Y., Chen S.-L. (2018). Miniature probe for in vivo optical- and acoustic-resolution photoacoustic microscopy. Opt. Lett..

